# Marmaricines A-C: Antimicrobial Brominated Pyrrole Alkaloids from the Red Sea Marine Sponge *Agelas* sp. *aff. marmarica*

**DOI:** 10.3390/md23020080

**Published:** 2025-02-12

**Authors:** Diaa T. A. Youssef, Areej S. Alqarni, Ameen M. Almohammadi, Turki Abujamel, Lamiaa A. Shaala

**Affiliations:** 1 Department of Natural Products, Faculty of Pharmacy, King Abdulaziz University, Jeddah 21589, Saudi Arabia; alqarniareej@gmail.com; 2 King Fahd Medical Research Center, King Abdulaziz University, Jeddah 21589, Saudi Arabia; tabujamel@kau.edu.sa; 3 Department of Pharmacy Practice, Faculty of Pharmacy, King Abdulaziz University, Jeddah 21589, Saudi Arabia; amalmohammadi@kau.edu.sa; 4 Department of Medical Laboratory Sciences, Faculty of Applied Sciences, King Abdulaziz University, Jeddah 21589, Saudi Arabia; 5 Suez Canal University Hospitals, Suez Canal University, Ismailia 41522, Egypt; lamiaa.elnady@med.suez.edu.eg

**Keywords:** Res Sea, *Agelas* sp. *aff. marmarica*, bioactive compounds, brominated pyrrole alkaloids, marmaricines A-C, antimicrobial activity

## Abstract

The Red Sea is the home of a rich diversity of sponge species with unique ecological adaptations that thrive in its saline, warm, and nutrient-poor waters. Red Sea sponges offer potential as sources of bioactive compounds and novel drugs. The organic extract of the Red Sea sponge *Agelas* sp. *aff. marmarica* was investigated for its antimicrobial constituents. Through bioassay-guided fractionation of the antimicrobial fraction of the extract on SiO_2_ and Sephadex LH-20, as well as HPLC purification, three bioactive compounds, marmaricines A-C (**1**–**3**), were isolated. Structural elucidation of the compounds was performed using 1D (^1^H and ^13^C) and 2D (COSY, HSQC, HMBC, and NOESY) NMR, as well as (+)-HRESIMS, leading to the identification of the compounds. The antimicrobial activities of the compounds were assessed through evaluation of their inhibition zones, MIC, MBC, and MFC, against Methicillin-Resistant *Staphylococcus aureus* (MRSA), *Escherichia coli*, and *Candida albicans*. Marmaricines A and B exhibited the strongest antibacterial effects against MRSA, with inhibition zones ranging from 14.00 to 15.00 mm, MIC values of 8 µg/mL, and MBC values of 16 µg/mL. In comparison, marmaracine C showed slightly weaker activity (inhibition zone: 12 mm, MIC: 16 µg/mL, MBC: 32 µg/mL). In terms of antifungal activity, marmaricines B and C demonstrated the greatest effect against *C. albicans*, with inhibition zones of 14–15 mm, MIC values of 8 µg/mL, and MFCs of 16 µg/mL. Interestingly, none of the compounds showed any inhibitory effect against *E. coli*. The results indicate that marmaricines A-C are selectively active against MRSA, and marmaricines B and C demonstrate potential against *C. albicans*, making them promising candidates for the development of novel antimicrobial agents targeting resistant pathogens.

## 1. Introduction

The Red Sea is renowned for its exceptional biodiversity [1] and endemism [2], hosting over 1000 fish species and more than 50 genera of hermatypic corals. Recent inventories have expanded the knowledge of the region’s biodiversity, identifying 635 polychaete, 79 ascidians, and 211 echinoderm species [2]. However, despite this remarkable richness, the Red Sea and the broader Arabian region remain relatively understudied compared to other worldwide coral reef systems, particularly with respect to sponge diversity [2,3]. One challenge in understanding the Red Sea ecosystem is that much of the existing research has been concentrated on a small stretch of coastline in the far northern Red Sea, specifically the Gulf of Eilat/Aqaba (~6 km in length) [4]. Nevertheless, the Red Sea is increasingly attracting attention from climate change researchers due to its distinctive environmental conditions, including high and variable water temperatures and high salinity levels (40.0 psu in the northern Red Sea) [5]. These unique conditions are believed to reflect the near-future state of oceans in other parts of the world [6]. While coral research in the Red Sea has received significant attention, knowledge of its sponge biodiversity remains limited in comparison to that for other regions in the world [7,8]. Early studies of Red Sea sponges primarily focused on cataloging regional biodiversity, with little exploration of their ecological roles or chemical properties. The reported sponge fauna of the Red Sea is primarily composed of species from the classes Demospongiae (225 species) and Calcarea (32 species). In contrast, much less is understood about Homoscleromorpha (2 species) and Hexactinellida (glass sponges), which are typically found in deeper waters and are currently represented by only two described species. It is likely that these two classes harbor many undiscovered species in the Red Sea [9]. Worldwide, marine sponges (Porifera) have become a primary focus of research due to their biologically active secondary metabolites. Red Sea sponges are particularly diverse in terms of their morphologies and the range of bioactive compounds they produce. These compounds not only play crucial roles in marine ecosystems, such as facilitating symbiosis with microorganisms, but also have potential pharmaceutical applications.

Marine sponges from the genus *Agelas* (class Demospongiae, order Agelasida, family Agelasidae) are among the most common sponges found in tropical and subtropical regions worldwide, with 36 valid species currently recognized. Ongoing research continues to expand our understanding of these species and their ecological and biochemical significance. Currently, only two species of *Agelas* have been reported in the Red Sea including *Agelas marmarica* and *Agelas mauritiana* [9]. The secondary metabolites isolated from *Agelas* sponges, since their initial discovery, represent a fascinating area of research that has driven significant advancements in the field of marine natural products [10]. Over five decades (1971–2021), more than 355 compounds have been reported from several members of the genus *Agelas* [10]. The largest producers of the compounds among the *Agelas* species are *A. oroides* (15%), *A. nakamurai* (13%), and *A. mauritiana* (11%), while the remainder was reported from unclassified *Agelas* species [10]. Members of the genus *Agelas* exhibit notable structural diversity in their pyrrole and terpenoidal alkaloids [11]. Following the isolation of specific bromopyrrole derivatives from *Agelas oroides* in 1971 [12] and the identification of agelasine, a quaternary 9-methyladenine derivative of an unidentified diterpene from *Agelas dispar* in 1975 [13], in addition to pyrrole and terpenoidal alkaloids, numerous bioactive metabolites of varying biogenetic origins, such as glycosphingolipids, sterols, carotenoids, and many others have been discovered within the genus *Agelas* [11,14,15,16,17,18]. Pyrrole alkaloids of this genus typically possess a backbone consisting of a bromo- or debromo-pyrrole-2-carboxamide structure, which is associated with various side chains and cyclic formations [19,20]. In contrast, the less common diterpene alkaloids primarily include those containing a 9-*N*-methyladeninium group (such as agelines, agelasines, and nemoechines) [21,22,23], as well as diterpenes related to hypotaurocyamine (for example, agelasidines) [24]. Secondary metabolites of the genus *Agelas* show a wide range of biological activities, including antimicrobial [25,26], antihistaminic [27], antimalarial [28], antileukemic [29], cytotoxic [26,30], antifouling [30,31], Na^+^,K^+^-adenosine triphosphatase (ATPase) inhibitory [21,24], and antiangiogenic matrix metalloproteinase inhibitory effects [32].

In our continuous effort to identify bioactive compounds from Red Sea marine sponges [33,34], we investigated the sponge *Agelas* sp. *aff. marmarica*. Bioassay-guided partitioning of the antimicrobial fraction of the organic extract of the sponge and final HPLC purification afforded three new brominated pyrrole-derived alkaloids, marmaricines A-C. The current study describes the isolation, structural elucidation, and antimicrobial activities of these compounds.

## 2. Results and Discussion

### 2.1. Purification of Compounds ***1***–***3***

**Figure 1:**
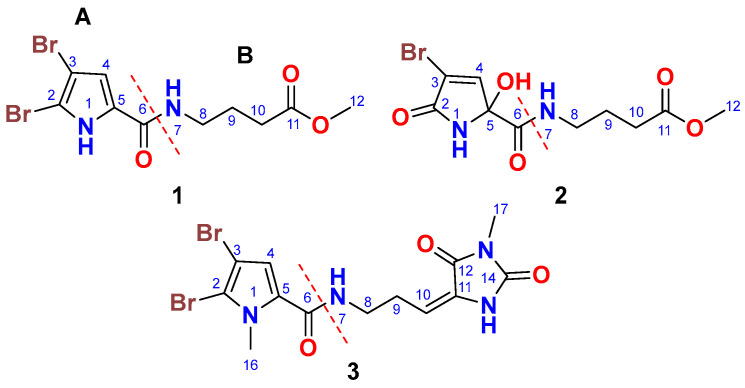
Chemical structures of compounds **1**–**3**.

Fractionation of the antimicrobial fraction of the organic extract of the Red Sea sponge *Agelas* sp. *aff. marmarica* on normal silica gel, Sephadex LH 20, and purification of the active fraction on HPLC afforded compounds **1**–**3** (Figure 1).

### 2.2. Structure of Compound ***1***

Compound **1** (Figure 1) was isolated as a yellowish powder with the molecular formula C_10_H_12_Br_2_N_2_O_3_, determined from the (+)-HRESIMS ion peak at *m*/*z* 388.9111 [M + Na]^+^. The presence of two bromine atoms in compound **1** was confirmed by the observation of three ion peaks at *m*/*z* 388.9, 390.9, and 392.9 in a 1:2:1 ratio. The structure of **1** was elucidated through the interpretation of its 1D and 2D NMR spectra. The ^13^C NMR spectrum showed signals for 10 carbon atoms (Table 1). Analysis of the ^13^C NMR spectrum, supported by the HSQC experiment, led to the assignment of five non-protonated carbons, one methine, three methylene groups, and one methyl group. The ^13^C NMR chemical shifts suggested the presence of two distinct parts (A and B) in compound **1,** including a 4,5-dibromo-2-carboxylic acid moiety (A) linked to a 4-amino-1-methylbutanoate (B) unit via an amidic linkage (CO-NH) at C-6/N*H*-7. The ^13^C NMR spectrum exhibited two carbonyl signals at δ_C_ 159.4 and 173.6, which were attributed to carboxamide (C-6) and carboxylic acid ester (C-11) groups, respectively. The ^1^H/^13^C signals at δ_H/C_ 12.65 (s) (N*H*-1), 104.9 (C, C-2), 98.2 (C, C-3), 6.90 (s)/112.9 (CH, C-4), and 128.6 (C, C-5) were consistent with the structure of an amidic derivative of 4,5-dibromo-pyrrole-2-carboxylic acid (substructure A).

**Figure 2:**
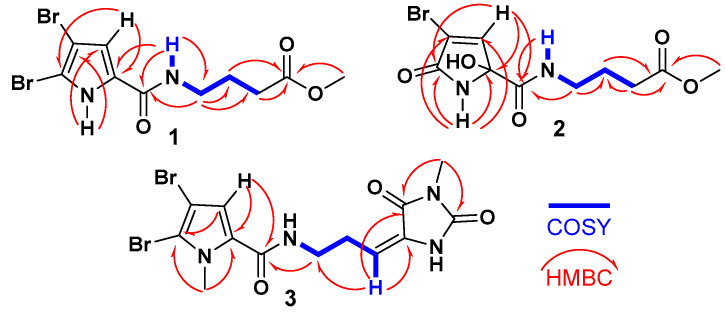
^1^H-^1^H COSY and ^1^H-^13^C HMBC correlations of compounds **1**–**3**.

The ^1^H-^1^H COSY spectrum revealed a single spin-coupling system from N*H*-7 through H_2_-8 to H_2_-10 (Figure 2). The signals at δ_H/C_ 8.14 (t, *J* = 5.6 Hz, N*H*-7), 3.21 (q, *J* = 6.5 Hz)/38.3 (CH_2_, C-8), 1.73 (quin., *J* = 7.0 Hz)/25.0 (CH_2_, C-9), and 2.34 (t, *J* = 7.4 Hz)/31.1 (CH_2_, C-10) confirmed this coupling system and this portion of the molecule.

Further HMBC correlations from the three-proton singlet at δ_H_ 3.57 (s) to C-11 (δ_C_ 173.6) supported the presence of a methyl ester group. HMBC couplings from H_2_-9 and H_2_-10 to C-11 (δ_C_ 174.9) further confirmed this assignment. The connectivity between the two parts of the compound was established through HMBC correlations (Figure 2) from H-4 (δ_H_ 6.90) to C-6 (δ_C_ 159.4), from N*H*-7 (δ_H_ 12.65) to C-6 (δ_C_ 159.4), and from H_2_-8 (δ_H_ 3.21) to C-6 (δ_C_ 159.4). Additional HMBC correlations from N*H*-1 (δ_H_ 12.65) to C-3 (δ_C_ 98.2) and C-4 (δ_C_ 112.9), and from H-4 (δ_H_ 6.90) to C-2 (δ_C_ 104.9) and C-5 (δ_C_ 128.6), confirmed the assignments of these carbons. Therefore, compound **1** was identified as 4-(4,5-dibromo-1H-pyrrole-2-carboxamido)-1-methylbutanoate. It is worth noting that compound **1** exists as a commercial product with the CAS number 1706460-02-7 and is sold by several chemical companies. In addition, a racemic mixture of a 9-hydroxy derivative of **1**, named agesasine B, was previously reported from the marine sponge *Agelas* spp., collected off the Kerama Islands, Okinawa [35]. Agesasine B was inactive against human cervical carcinoma (HeLa), lung carcinoma (A549), and breast cancer (MCF7) cell lines, with IC_50_ values > 100 µM [35]. Recently, both enantiomers of agesasine B were synthesized [36]. The (*S*)-agesasine B displayed moderate cytotoxic activity against breast cancer cell line (MCF7), with an IC_50_ value of 18 μM, while the (*R*)-agesasine B was inactive against this cell line. When tested against liver cancer cells (HEPG2), (*R*)-agesasine B was weakly active with IC_50_ of 52 μM, while (*S*)-agesasine B was inactive. Finally, both enantiomers were highly weakly active towards colorectal adenocarcinoma (CACO2) (IC_50_ = 72–74 μM) [36]. Conclusively, this is the first report of the isolation of compound **1** from a natural source, making it a newly identified natural product. The generic name marmaricine A was given to compound **1**.

### 2.3. Structure of Compound ***2***

Compound **2** (Figure 1) was isolated as an optically inactive yellowish powder ([α]_D_ 0°, c 0.10, MeOH) with the molecular formula C_10_H_13_BrN_2_O_5_, as determined from the (+)-HRESIMS ion peak at *m*/*z* 342.9906 [M + Na]^+^. The observation of two ion peaks at *m*/*z* 342.9 and 344.9 in a 1:1 ratio confirmed the presence of a single bromine atom in compound **2**. The structure of compound **2** was determined through the analysis of its 1D and 2D NMR spectra. The ^13^C NMR spectrum showed signals for 10 carbon atoms (Table 1). Interpretation of the ^13^C NMR data, combined with the HSQC experiment, allowed for the assignment of the carbons as five non-protonated carbons, i.e., one methine, three methylene groups, and one methyl group. The COSY, ^13^C NMR, HSQC, and HMBC data facilitated the identification of two main parts (A and B) in compound **2**, including a 4-bromo-2-hydroxy-5-oxo-2,5-dihydro-1H-pyrrole-2-carboxamide moiety (A) linked to a 4-amino-1-methylbutanoate unit (B) via an amidic bond (C-6/N*H*-7).

When compared to compound **1**, which contains a 4,5-dibromo-1H-pyrrole-2-carboxamide moiety as part A, compound **2** features a 4-bromo-2-hydroxy-5-oxo-2,5-dihydro-1H-pyrrole-2-carboxamide moiety. The assignment of this substructure in compound **2** was supported by the ^1^H/^13^C NMR signals at δ_H/C_ 9.05 (N*H*-1), 167.5 (C, C-2), 120.3 (C, C-3), 7.24 (d, *J* = 1.6 Hz)/147.0 (CH, C-4), 87.8 (C, C-5), and 167.3 (C, C-6). HMBC correlations (Figure 2) from N*H*-1 to C-2, C-3, C-5, and C-6, as well as from H-4 to C-2 and C-5, supported this assignment. The chemical shifts of the ^1^H and ^13^C NMR signals for the second part of compound **2** (B) are like those in compound **1**, suggesting that the same substructure is present in both molecules (Table 1). The connection between the two parts of compound **2** was further confirmed by HMBC correlations (Figure 2) from H-4 to C-6, from N*H*-7 to C-6, and from H_2_-8 to C-6 (δ_C_ 167.3). The racemic nature of compound **2** was confirmed by the lack of any optical activity and the absence of any Cotton effects (CE) in the experimental ECD spectrum. Therefore, compound **2** was determined to be a racemic mixture and assigned as (±)-4-(4-bromo-2-hydroxy-5-oxo-2,5-dihydro-1H-pyrrole-2-carboxamido)-1-methylbutanoate. Compound **2** is reported here as a new natural product and named marmaricine B.

### 2.4. Structure of Compound ***3***

Compound **3** (Figure 1) was purified and obtained as a yellowish powder with the molecular formula C_13_H_14_Br_2_N_4_O_3_, as indicated by the (+)-HRESIMS ion peak at *m*/*z* 454.9327 [M + Na]^+^, which suggests the presence of eight degrees of unsaturation. The detection of three ion peaks at *m*/*z* 454.9, 456.9, and 458.9 in a 1:2:1 ratio further corroborates the dibrominated nature of compound **3**. The structure for compound **3** was determined through analysis of its 1D (^1^H and ^13^C) and 2D (COSY, HSQC, HMBC, and NOESY) NMR spectra. The NMR data (Table 1) supports the presence of two substructures (A and B). Except for the absence of the N*H*-1 signal, compound **3** exhibited similar ^1^H and ^13^C NMR signals to the substructure A of compound **1**. Notably, the ^1^H/^13^C NMR signals at 3.90 (3H, s, H_3_-16)/36.4 (CH_3_, C-16) in the ^1^H and ^13^C NMR spectra of compound **3**, along with the HMBC correlations (Figure 2) from H_3_-16 (δ_H_ = 3.90) to C-2 (δ_C_ = 111.9) and C-5 (δ_C_ = 129.4), and from H-4 (δ_H_ = 6.24) to C-2, C-5, and C-6 (δ_C_ = 161.4), support the assignment of substructure A as the 4,5-dibromo-1-methyl-1H-pyrrole-2-carboxamide moiety.

**Figure 3:**
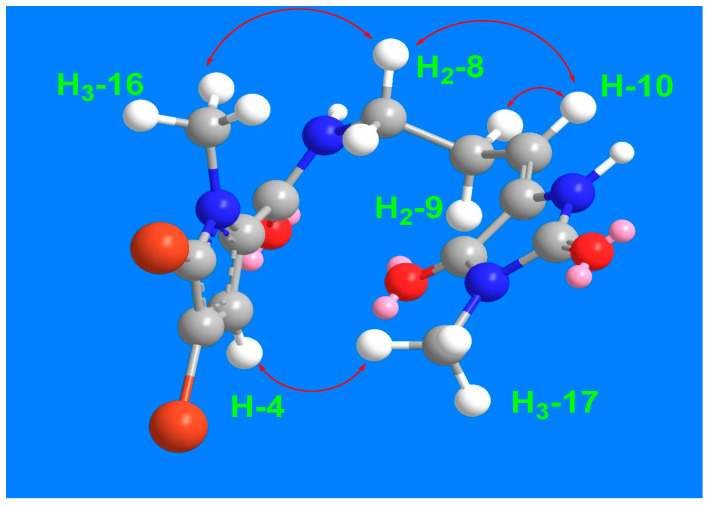
Significant ^1^H-^1^H NOESY correlations of compound **3**.

The remaining signals for compound **3** were attributed to the 5-(3-aminopropylidene)-3-methylimidazolidine-2,4-dione moiety, based on the ^1^H and ^13^C NMR signals at δ_H/C_ 3.45 (t, *J* = 7.5 Hz)/39.2 (CH_2_, C-8), 2.61 (q, *J* = 7.5 Hz)/28.8 (CH_2_, C-9), 6.24 (q, *J* = 7.5 Hz)/120.5 (CH, C-10), 130.0 (C, C-11), 163.1 (C, C-12), 154.0 (C, C-14), and 3.20 (3H, s, H_3_-17)/26.5 (CH_3_, C-17). This assignment is further validated by the COSY correlations (Figure 2) from H_2_-8 to H-10 and the HMBC correlations (Figure 2) from H-10 to C-8 (δ_C_ = 39.2), C-11 (δ_C_ = 130.0), and C-12 (δ_C_ = 163.1), as well as from CH_3_-17 to C-12 (δ_C_ = 163.1) and C-14 (δ_C_ = 154.0). The interconnection between the substructures in compound **3** is also supported by the HMBC correlation (Figure 2) from H_2_-8 to C-6. Additionally, the assignment of the ^1^H and ^13^C NMR signals for substructure B was confirmed through the COSY (Figure 2) and HMBC correlations. The *E* configuration at Δ^10,11^ is supported by NOESY correlations between H-10 and H_2_-8 and between H_3_-17 and H-4, as well as between H_2_-8 and H_3_-16 (Figure 3).

Previously, compound **3** was synthesized as a mixture of *E* and *Z* isomers in a 1:3 ratio [37]. To compare the ^13^C NMR data of compound **3** with the previously published data of the synthetic compound, the NMR spectra of **3** were acquired in DMSO-*d*_6_. The ^13^C NMR data for compound **3** closely resemble those reported for the *E* isomer of (*E/Z*)-4,5-dibromo-*N*-[3-(1-methyl-2,5-dioxo-imidazolidin-4-ylidene)propyl]-1-methylpyrrole-2-carboxamide [37]. In conclusion, compound **3** was assigned as (*E*)-4,5-dibromo-1-methyl-*N*-(3-(1-methyl-2,5-dioxoimidazolidin-4-ylidene)propyl)-1H-pyrrole-2-carboxamide and is being reported here as a new natural product, named marmaricine C.

The antimicrobial activities of marmaricines A-C were evaluated using the inhibition zones, minimum inhibitory concentration (MIC), minimum bactericidal concentration (MBC), and minimum fungicidal concentration (MFC) against Methicillin-Resistant *S. aureus* (MRSA), *E. coli*, and *C. albicans*. Marmaricines A and B showed the highest antibacterial activity against MRSA, with inhibition zones ranging from 14.00 to 15.00 mm, MIC values of 8 µg/mL, and MBC values of 16 µg/mL (Table 2), indicating potent activity. In comparison, marmaracine C showed weaker antibacterial effects, with a 12 mm inhibition zone, an MIC of 16 µg/mL, and an MBC of 32 µg/mL, highlighting its lower potency against MRSA. When tested against *C. albicans*, marmaricines B and C demonstrated significant antifungal activity, with inhibition zones of 14–15 mm, MIC values of 8 µg/mL, and MFC values of 16 µg/mL (Table 2). However, none of the compounds showed activity against *E. coli*, indicating that they are more effective against Gram-positive bacteria and fungi. These results underline the selective antimicrobial properties of marmaricines A-C, particularly their effectiveness against MRSA and *C. albicans*. Marmaricines A and B are strong candidates for further development as antibacterial agents, especially against resistant strains like MRSA, while marmaracine C, though less potent, still holds potential. The antifungal activity of marmaricines B and C against *C. albicans* also makes them promising agents for fungal infections. Further, the lack of activity against *E. coli* indicates selectivity of these compounds against MRSA and *C. albicans*.

## 3. Materials and Methods

### 3.1. General Experimental Procedures

Optical rotations were recorded using a JASCO DIP-370 digital polarimeter (Jasco Co., Tokyo, Japan) at 25 °C, with measurements taken at the sodium D line (589 nm). The (+)-LRESIMS mass spectra of the compounds were evaluated using an Agilent 1200 system connected to an Agilent 6320 Ion Trap LC-ESI-M. The HPLC included a solvent delivery module, a quaternary pump, an auto sampler, and a column compartment (Agilent Technologies Deutschland GmbH, Waldbronn, Germany). The column effluent was connected to an Agilent 6320 Ion Trap LC-ESI-MS. The column heater was set at 25 ± 2 °C, and the HPLC system control and data processing were carried out using ChemStation (Rev. B.01.03-SR2, 204) and 6300 Series Trap Control version 6.2, Build No. 62.24 (Bruker Daltonik GmbH, Bremen, Germany). The compounds were run on an Agilent Zorbax Eclipse XDB-C18 column (250 × 4.6 mm, 5 μm particle diameter), protected with an Agilent Zorbax XDB-C18 pre-column. The mobile phase was programmed to be a gradient from 10% acetonitrile to 0% acetonitrile in 30 min, at a flow rate of 0.5 mL/min. The ESI-MS ion trap conditions included a smart target ion up to 1500 *m*/*z*, a nebulizer at 36 psi, dry gas at 12 L/min, and dry temperature at 350 °C. The (+)-HRESIMS mass spectral data were obtained with a Micromass Q-tof equipped with a lockspray mass spectrometer, using Leucine Enkaphalin at *m*/*z* 556.2771 [M + H]^+^ as a reference mass. One-dimensional and two-dimensional NMR spectra (chemical shifts in ppm and coupling constants in Hz) were acquired on Bruker Avance DRX 800 MHz (800 MHz for ^1^H and 200 MHz for ^13^C) or 500 MHz (500 MHz for ^1^H and 125 MHz for ^13^C) spectrometers (Bruker, Rheinstetten, Germany), using DMSO-*d*_6_ or CD_3_OD as the solvent. HPLC separation was carried out on a C18 column (Atlantis^®^, 150 × 4.6 mm, 2.5 μm, Waters Corporation, Milford, MA, USA), with a CH_3_CN:H_2_O gradient as the mobile phase, monitored at 220 nm, and a flow rate of 2.0 mL/min.

### 3.2. Biological Materials

**Figure 4:**
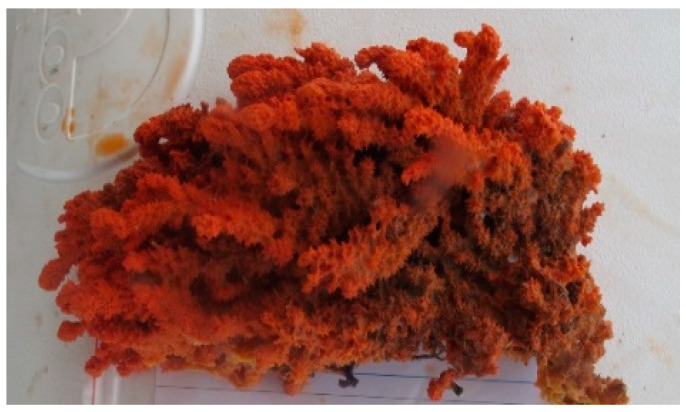
A photograph of the Red Sea sponge *Agelas* sp. *aff. marmarica*.

The Red Sea sponge *Agelas* sp. *aff. marmarica* (Figure 4) was collected off the Saudi Red Sea coast (N 021°39′17.5′′, E 038°52′26.3′′). The sponge belongs to kingdom: Animalia, phylum: Porifera, class: Demospongiae, subclass: Heteroscleromorpha, order: Agelasida, family: Agelasidae, genus: *Agelas*, and species: *Agelas* sp. *aff. marmarica*. The sponge was kindly identified by Rob van Soest. A specimen of the sponge was kept at the collection of the Naturalis Biodiversity Center at Leiden, The Netherlands, under registration number RMNHPOR 9165. Another specimen was stored at the Red Sea Invertebrates Collection at King Abdulaziz University, under code No. DY-16.

### 3.3. Purification of the Compounds

The freeze-dried sponge materials (0.35 Kg) were macerated in a mixture of CH_2_Cl_2_:CH_3_OH (1:1) (3 × 2000 mL) at room temperature. The combined extracts were dried under reduced pressure to yield a brown residue. The dried residue (17.5 g) was subjected to partitioning on a VLC silica gel column using n-hexane-CH_2_Cl_2_-MeOH gradients, affording 12 main fractions (Fr. 1–12). The antimicrobial fraction eluted with 100% CH_2_Cl_2_, Fr. 6 (0.41 g) (inhibition zone = 8 mm against *C. albicans*) was subjected to partitioning on Sephadex LH-10 using MeOH to afford five factions (Fr. A-E). The antimicrobial fraction (Fr. C) (126 mg) (inhibition zone = 10 mm against *C. albicans*) was purified on a reversed-phase HPLC column (XDB-C18, 250 × 9.4 mm 5 µm, Agilent) using CH_3_CN:H_2_O gradients at 2 mL/min, starting from 20% CH_3_CN to 0% CH_3_CN in 50 min to yield compounds **1** (2.5 mg, t*_R_* = 11 min), **2** (3.9 mg, t*_R_* = 20.5 min), and **3** (4.7 mg, t*_R_* = 17.5 min).

### 3.4. Spectral Data of ***1***–***3***

#### 3.4.1. Marmaricine A (**1**)

Yellowish powder; HRESIMS *m*/*z* 388.9111 (calcd for C_10_H_12_Br_2_N_2_O_3_Na [M + Na]^+^, 388.9106); ^1^H NMR (800 MHz, DMSO-*d*_6_): 12.65 (1H, s, N*H*-1), 6.90 (1H, s, H-4), 8.14 (1H, t, *J* = 5.6 Hz, N*H*-7), 3.21 (2H, q, *J* = 6.5 Hz, H_2_-8), 1.73 (2H, quin., *J* = 7.1 Hz, H_2_-9), 2.34 (2H, t, *J* = 7.4 Hz, H_2_-10), 3.57 (3H, s, H_3_-12); ^13^C NMR (200 MHz, DMSO-*d*_6_): 104.9 (C, C-2), 98.2 (C, C-3), 112.9 (CH, C-4), 128.6 (C, C-5), 159.4 (C, C-6), 38.3 (CH_2_, C-8), 25.0 (CH_2_, C-9), 31.1 (CH_2_, C-10), 173.6 (C, C-11), 51.7 (CH_3_, C-12).

#### 3.4.2. Marmaricine B (**2**)

Yellowish powder; HRESIMS *m*/*z* 342.9906 (calcd for C_10_H_13_BrN_2_O_5_Na [M + Na]^+^, 342.9900); ^1^H NMR (800 MHz, DMSO-*d*_6_): 9.05 (1H, s, N*H*-1), 7.24 (1H, t, *J* = 1.6, H-4), 8.21 (1H, t, *J* = 5.9 Hz, N*H*-7), 3.07 (2H, q, *J* = 7.8 Hz, H_2_-8), 1.65 (2H, quin., *J* = 7.2 Hz, H_2_-9), 2.27 (2H, t, *J* = 7.5 Hz, H_2_-10), 3.55 (3H, s, H_3_-12); ^13^C NMR (200 MHz, DMSO-*d*_6_): 167.5 (C, C-2), 120.3 (C, C-3), 147.0 (CH, C-4), 87.8 (C, C-5), 167.3 (C, C-6), 38.8 (CH_2_, C-8)**,** 24.7 (CH_2_, C-9), 31.0 (CH_2_, C-10), 173.6 (C, C-11), 51.7 (CH_3_, C-12).

#### 3.4.3. Marmaricine C (**3**)

Yellowish powder; [α]_D_ 0° (c 0.1, MeOH); HRESIMS *m*/*z* 454.9327 (calcd for C_13_H_14_Br_2_N_4_O_3_Na [M + Na]^+^, 454.9324); NMR data: ^1^H NMR (500 MHz, CD_3_OD): 6.82 (1H, s, H-4), 3.45 (2H, t, *J* = 7.5 Hz, H_2_-8), 2.61 (2H, q, *J* = 7.5 Hz, H_2_-9), 6.24 (1H, t, *J* = 7.5 Hz, H-10), 3.90 (3H, s, H_3_-16), 3.20 (3H, s, H_3_-17); ^13^C NMR (125 MHz, CD_3_OD): 111.9 (C, C-2), 99.2 (C, C-3), 116.1 (CH, C-4), 129.4 (C, C-5), 161.4 (C, C-6), 39.2 (CH_2_, C-8)**,** 28.8 (CH_2_, C-9), 120.5 (CH, C-10), 130.0 (C, C-11), 163.1 (C, C-12), 154.0 (C, C-14), 36.4 (CH_3_, C-16)., 26.5 (CH_3_, C-17); ^1^H NMR (800 MHz, DMSO-*d*_6_): 7.08 (s, H-4), 8.19 (t, *J* = 7.6 Hz, H-7), 3.26 (q, *J* = 7.6, H_2_-8), 2.37 (q, *J* = 7.5, H_2_-9), 5.96 (t, *J* = 7.3 Hz, H-10), 10.93 (brs, H-15), 3.87 (s, H_3_-16), 3.03 (s, H_3_-17); ^13^C NMR (200 MHz, DMSO-*d*_6_): 110.3, 96.7, 114.8, 127.8, 159.7, 38.2, 25.9, 109.9, 128.9, 163.0, 153.5, 35.2, 23.6.

### 3.5. Antimicrobial Activities of the Compounds

#### 3.5.1. Disk Diffusion Assay

The in vitro antimicrobial activity was evaluated using the disc diffusion method, as previously described [38,39,40,41,42,43]. A variety of test microorganisms were employed, including *Staphylococcus aureus* (ATCC 43300, methicillin-resistant), *Escherichia coli* (ATCC 35218), and *Candida albicans* (ATCC 76615). Each microorganism was inoculated to a turbidity matching a 0.5 McFarland standard and evenly streaked over the surface of Muller–Hinton agar plates using sterile swabs. Sterile 6 mm filter paper discs were impregnated with 50 μg of each compound and placed onto the inoculated agar. The plates were then incubated at 37 °C for 24 h. Solvent control discs were included to assess any potential solvent effects. Ciprofloxacin (5 μg/disc) served as the antibacterial reference, while clotrimazole (10 μg/disc) was used as the antifungal reference. The antimicrobial activity of each compound was determined by measuring the diameter of the inhibition zones in millimeters. The experiment was performed in duplicate, and the mean diameter of each inhibition zone was recorded.

#### 3.5.2. Evaluation of the Minimum Inhibitory Concentrations (MICs)

The minimum inhibitory concentration (MIC) of the compounds was determined using the broth microdilution method in accordance with the CLSI standards (CLSI M02 and CLSI M07) [38,39]. Two-fold serial dilutions of the compounds were prepared in Muller–Hinton broth (Sigma-Aldrich, St. Louis, MO, USA), with final concentrations ranging from 1.0 to 1000 μg/mL. A volume of 100 μL from each dilution was added to the wells of a 96-well plate in duplicate, followed by 100 μL of the bacterial inoculum. The inoculum was prepared by diluting 0.5 McFarland suspensions 150-fold in Muller–Hinton broth, achieving a density of 5 × 10^5^; CFU/mL. Growth control (without compound) and sterility control (without compound and inoculum) were also included. Ciprofloxacin and clotrimazole, used as a reference antibiotic and antifungal, respectively, was tested at concentrations ranging from 0.125 to 64 μg/mL. The prepared plates were incubated at 35 ± 1 °C for 18 ± 1 h. MIC was defined as the lowest concentration of the compound that inhibited bacterial growth, measured spectrophotometrically using a microplate reader (OD600, Boeco BMR-100) (Boeckel + Co (GmbH + Co), Hamburg, Germany)

#### 3.5.3. Determination of the Minimum Bactericidal Concentrations (MBCs)

After incubating the microplates and determining the MIC values of the compounds against MRSA, the MBCs of the compounds were determined by sub-culturing 25 μL of the test solution onto Mueller–Hinton agar and were considered as the lowest concentration that killed 99.99% of the initial inoculum.

#### 3.5.4. Assessment of the Minimum Fungicidal Concentrations (MFCs)

After incubating the microplates and determining the MIC values against *C. albicans*, the minimum fungicidal concentrations (MFCs) of the compounds were assessed. To do this, a 20 µL aliquot of the culture medium was taken from a well where no growth (no turbidity) was observed and plated onto Sabouraud dextrose agar plates. These plates were then incubated at 37 °C for 24–48 h. The MFC value was defined as the lowest concentration at which no more than one colony grew.

## 4. Conclusions

The organic extract derived from the Red Sea sponge *Agelas* sp. *aff. marmarica* has demonstrated remarkable promise as a source of antimicrobial agents. This study undertook a detailed investigation aimed at identifying and characterizing the bioactive constituents of this sponge through a rigorous bioassay-guided fractionation process. Utilizing chromatographic techniques, including silica gel column chromatography, Sephadex LH-20, and HPLC, three distinct bioactive compounds were isolated, designated as marmaricines A-C (**1**–**3**). The structural characterization of these compounds was carried out by interpreting their 1D and 2D NMR and (+)-HRESIMS. These methodologies provided comprehensive insights into the molecular structures of the isolated compounds, facilitating the identification of their unique chemical features. Marmaricines A and B demonstrated the most potent antibacterial effects against methicillin-resistant *S. aureus* (MRSA), with inhibition zones of 14.00–15.00 mm, MIC values of 8 µg/mL, and MBC values of 16 µg/mL. Marmaracine C, while exhibiting weaker antibacterial activity, also showed efficacy against MRSA (inhibition zone: 12 mm, MIC: 16 µg/mL, MBC: 32 µg/mL). In addition, marmaricines B and C showed significant antifungal activity against *C. albicans*, with inhibition zones of 14–15 mm, MICs of 8 µg/mL, and MFCs of 16 µg/mL. Notably, none of the compounds demonstrated activity against *E. coli*. These findings suggest that marmaricines A-C possess selectivity towards MRSA and *C. albicans* and are promising candidates for developing new antimicrobial agents, particularly against resistant pathogens such as MRSA and *C. albicans*.

Future investigations should focus on the isolation of larger amounts of the natural marmaricines and the preparation of related compounds through derivatization or total synthesis to understand the mechanisms of action of this class of compounds and their related analogs, particularly their specificity towards MRSA and *C. albicans*. Expanding the antimicrobial spectrum of these compounds to include other resistant pathogens would be an important next step. Further pharmacological studies, including in vivo testing and toxicity assessments, are essential to evaluate the clinical potential of these compounds. Additionally, exploring the possible synergistic effects of marmaricines in combination with existing antibiotics could enhance their efficacy and provide novel therapeutic strategies for combating drug-resistant infections.

## Figures and Tables

**Table 1:** NMR data of compounds **1**–**3**.

Position	δ_C_, Type	δ_H_ (Mult., *J* in Hz)	δ_C_, Type	δ_H_ (Mult., *J* in Hz)	δ_C_, Type	δ_H_ (mult., *J* in Hz)
	1 ^1^		2 ^1^		3 ^2^	
1		12.65 (s)		9.05 (brs)		
2	104.9, C		167.5, C		111.9, C	
3	98.2, C		120.3, C		99.2, C	
4	112.9, CH	6.90 (s)	147.0, CH	7.24 (t, 1.6)	116.1, CH	6.82 (s)
5	128.6, C		87.8, C		129.4, C	
6	159.4, C		167.3, C		161.4, C	
7		8.14 (t, 5.6)		8.21 (t, 5.9)		
8	38.3, CH_2_	3.21 (q, 6.5)	38.8, CH_2_	3.07 (q, 7.8)	39.2, CH_2_	3.45 (t, 7.5)
9	25.0, CH_2_	1.73 (quin., 7.1)	24.7, CH_2_	1.65 (quin., 7.2)	28.8, CH_2_	2.61 (q, 7.5)
10	31.1, CH_2_	2.34 (t, 7.4)	31.0, CH_2_	2.27 (t, 7.5)	120.5, CH	6.24 (t, 7.5)
11	173.6, C		173.6, C		130.0, C	
12	51.7, CH_3_	3.57 (s)	51.7, CH_3_	3.55 (s)	163.1, C	
13						
14					154.0, C	
16					36.4, CH_3_	3.90 (s)
17					26.5, CH_3_	3.20 (s)

^1^ Acquired at 800 MHz for ^1^H and 200 MHz for ^13^C NMR spectra in (DMSO-*d*_6_). ^2^ Acquired at 500 MHz for ^1^H and 125 MHz for ^13^C NMR spectra in (CD_3_OD).

**Table 2:** Antimicrobial activities of compounds **1**–**3**.

Compound	MRSA			*C. albicans*		
	Inhibition Zone (mm)	MIC (μg/mL)	MBC (μg/mL)	Inhibition Zone (mm)	MIC (μg/mL)	MFC (μg/mL)
**1**	14	8	16	NI ^4^	NT	NT
**2**	15	8	16	15	8	16
**3**	12	16	32	14	8	16
Ciprofloxacin ^1^	15	0.25	1.0	NT	NT	NT
Clotrimazole ^2^	NT ^3^	NT	NT	18	0.125	0.5

^1^ Positive antibacterial control (5 µg/disc); ^2^ positive antifungal control (10 μg/disc); ^3^ not tested; ^4^ no inhibition.

## Data Availability

All data related to this manuscript are available in the manuscript and its related Supplementary Materials.

## Supplementary Materials

The following supporting information can be downloaded at: www.mdpi.com/article/10.3390/md23020080/s1, Figures S1–S24: (+)-LRESIMS, (+)-HRESIMS, 1D (^1^H and ^13^C), and 2D (COSY, HSQC, HMBC, NOESY) NMR spectra of compounds **1**–**3**.
