# Supplementary material for: Marmaricines A-C: Antimicrobial Brominated Pyrrole Alkaloids from the Red Sea Marine Sponge Agelas sp. aff. marmarica
Source: Mar Drugs. 2025 Feb 12;23(2):80. doi: 10.3390/md23020080 (PMC11857417; doi:10.3390/md23020080)
Supplement: Supplementary file 1 [file marinedrugs-23-00080-s001.zip › marinedrugs-3444674-supplementary-Figures S1-S24.pdf]

Supplementary Information

(+)-LRESIMS Spectrum of Compound **1**	S1
(+)-HRESIMS Spectrum of Compound **1**	S2
^{1}H NMR Spectrum of Compound **1** (DMSO-d_6)	S3
^{13}C NMR Spectrum of Compound **1** (DMSO-d_6)	S4
^{1}H-^{1}H COSY Spectrum of Compound **1** (DMSO-d_6)	S5
HSQC Spectrum of Compound **1** (DMSO-d_6)	S6
HMBC Spectrum of Compound **1** (DMSO-d_6)	S7
(+)-LRESIMS Spectrum of Compound **2**	S8
(+)-HRESIMS Spectrum of Compound **2**	S9
^{1}H NMR Spectrum of Compound **2** (DMSO-d_6)	S10
^{13}C NMR Spectrum of Compound **2** (DMSO-d_6)	S11
^{1}H-^{1}H COSY Spectrum of Compound **2** (DMSO-d_6)	S12
HSQC Spectrum of Compound **2** (DMSO-d_6)	S13
HMBC Spectrum of Compound **2** (DMSO-d_6)	S14
(+)-LRESIMS Spectrum of Compound **3**	S15
(+)-HRESIMS Spectrum of Compound **3**	S16
^{1}H NMR Spectrum of Compound **3** (CD_3OD)	S17
^{13}C NMR Spectrum of Compound **3** (CD_3OD)	S18
^{1}H-^{1}H COSY Spectrum of Compound **3** (CD_3OD)	S19
HSQC Spectrum of Compound **3** (CD_3OD)	S20
HMBC Spectrum of Compound **3** (CD_3OD)	S21
^{1}H-^{1}H NOESY Spectrum of Compound **3** (CD_3OD)	S22
^{1}H NMR Spectrum of Compound **3** (DMSO-d_6)	S23
^{13}C NMR Spectrum of Compound **3** (DMSO-d_6)	S24

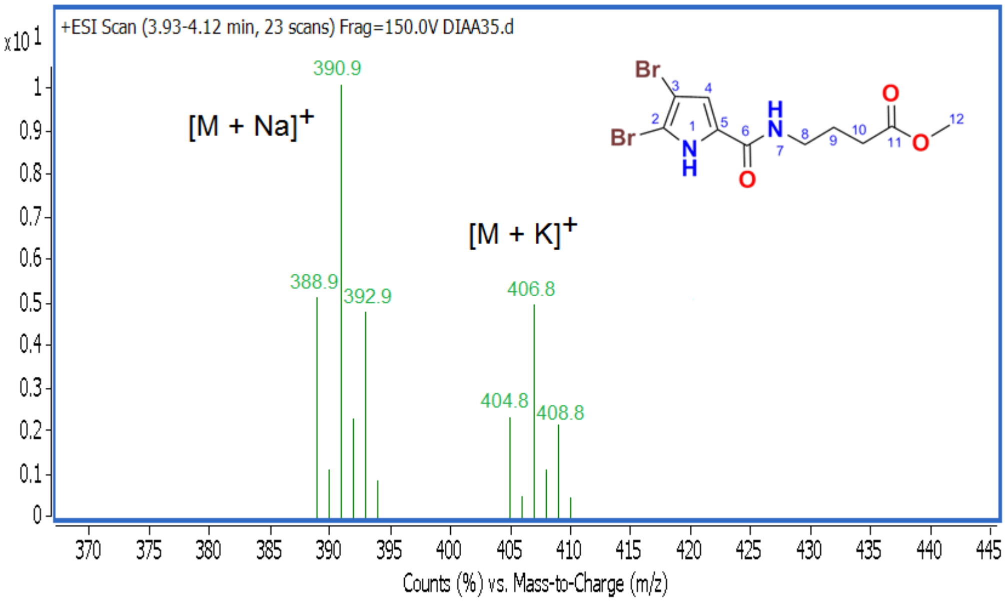

(+)-LRESIMS Spectrum of Compound **1**.

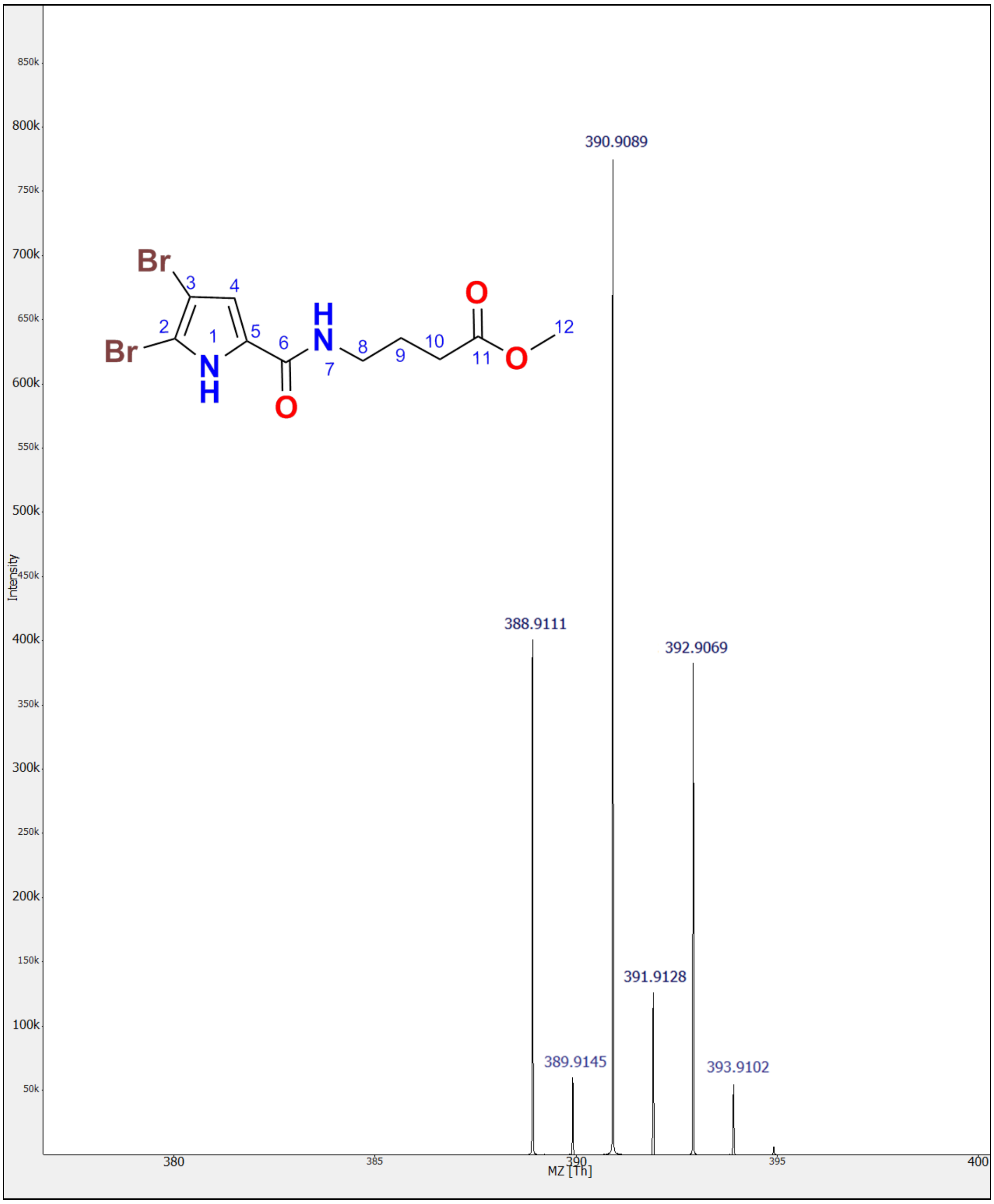

(+)-HRESIMS Spectrum of Compound **1**.

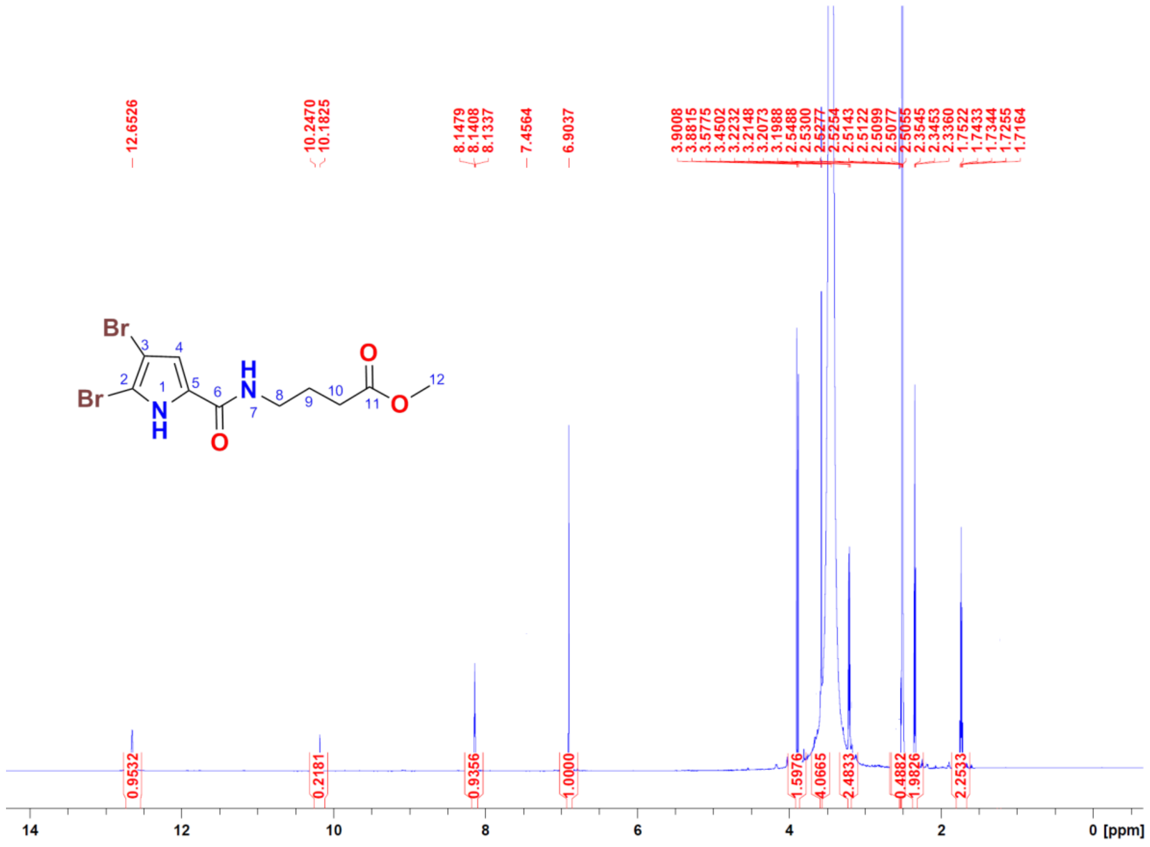

^{1}H NMR Spectrum of Compound **1** (DMSO-d_6).

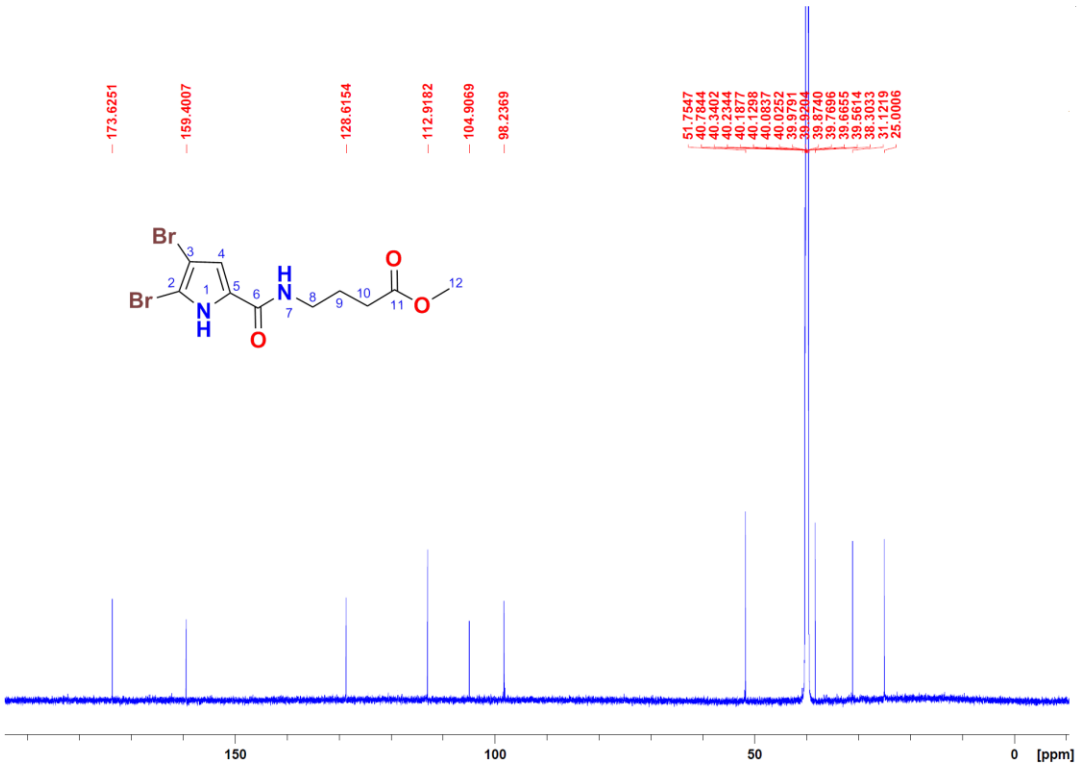

^{13}C NMR Spectrum of Compound **1** (DMSO-d_6).

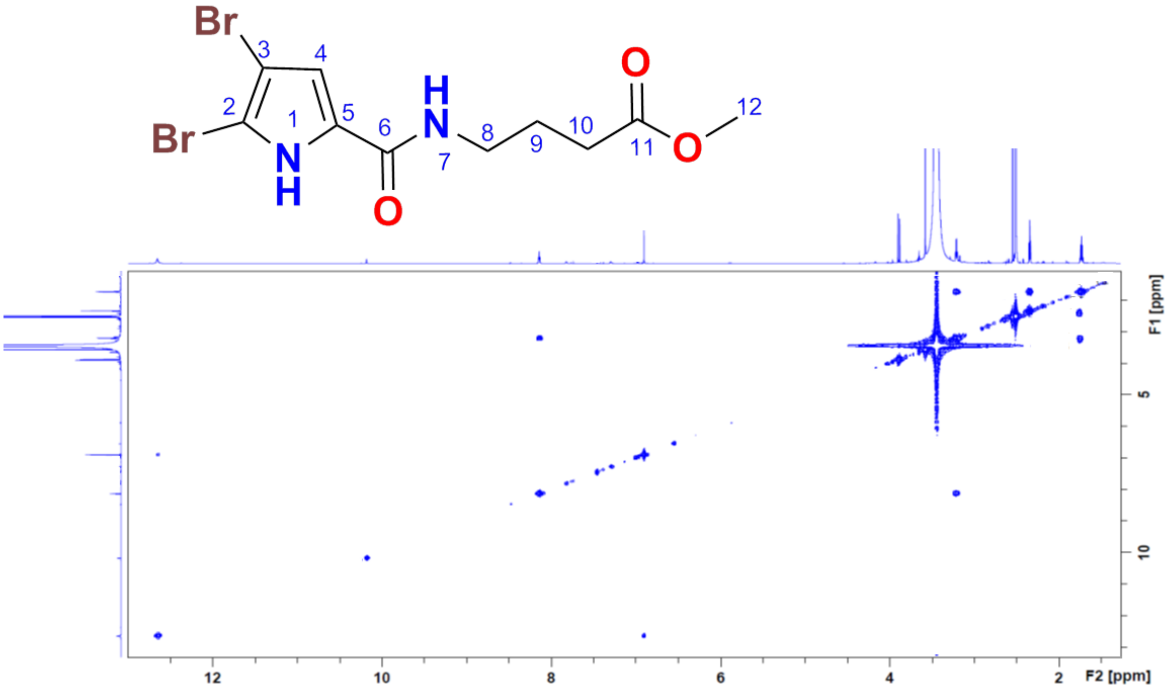

^{1}H-^{1}H COSY NMR Spectrum of Compound **1** (DMSO-d_6).

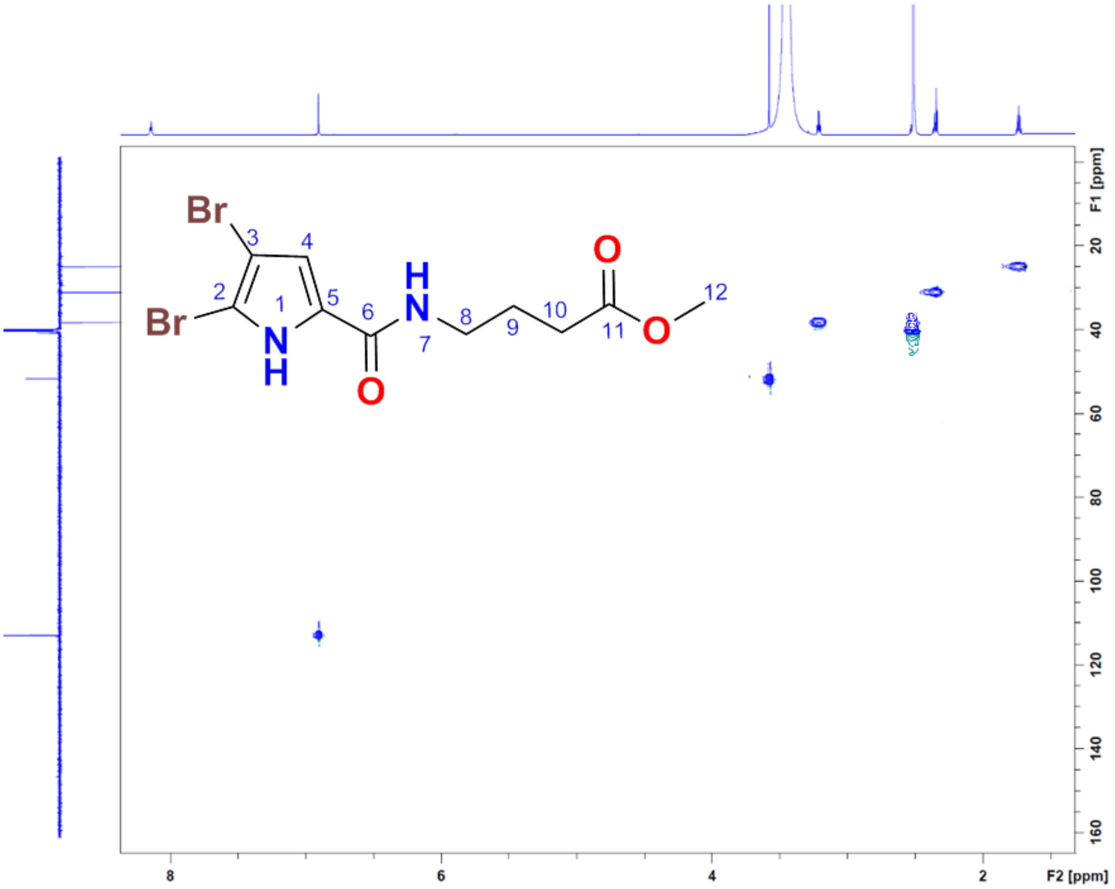

HSQC Spectrum of Compound **1** (DMSO-d_6).

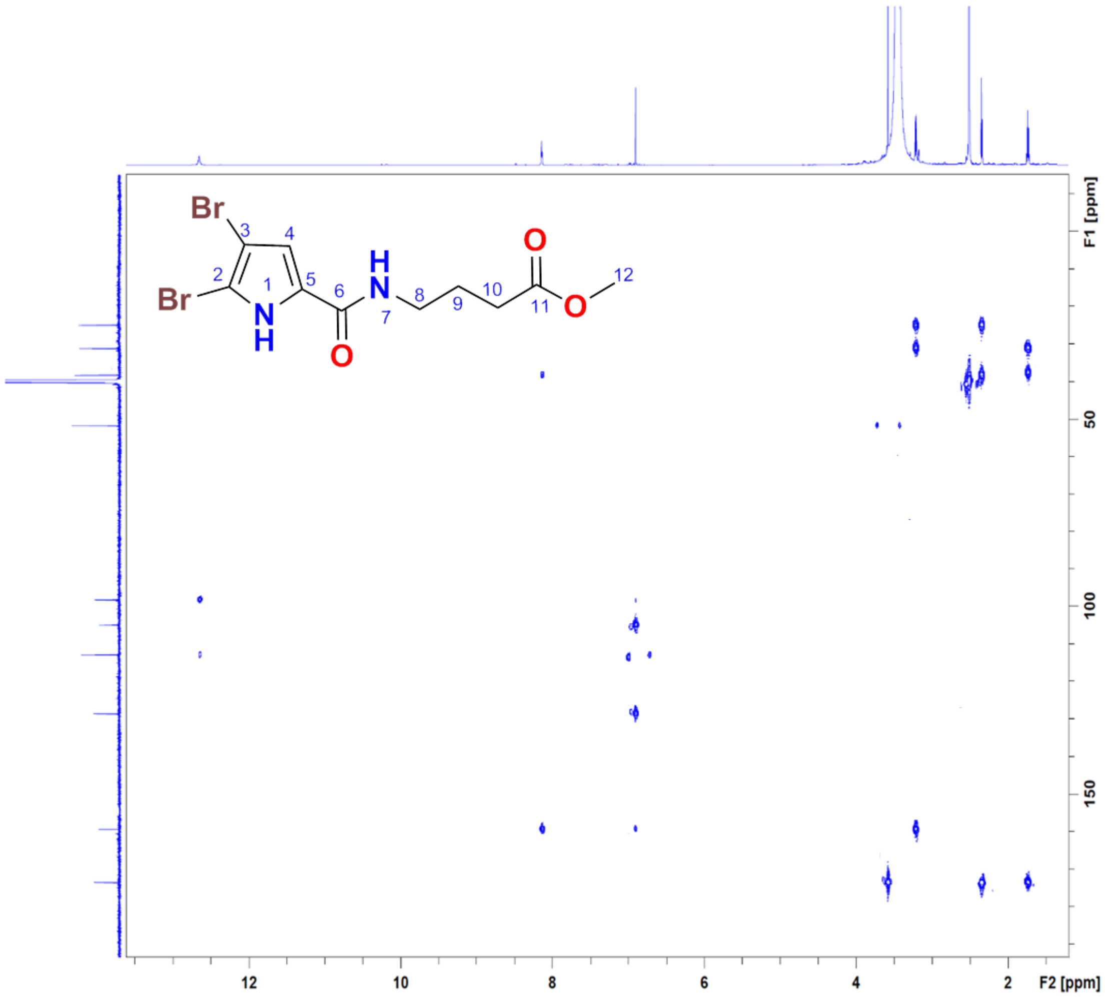

HMBC Spectrum of Compound **1** (DMSO-d_6).

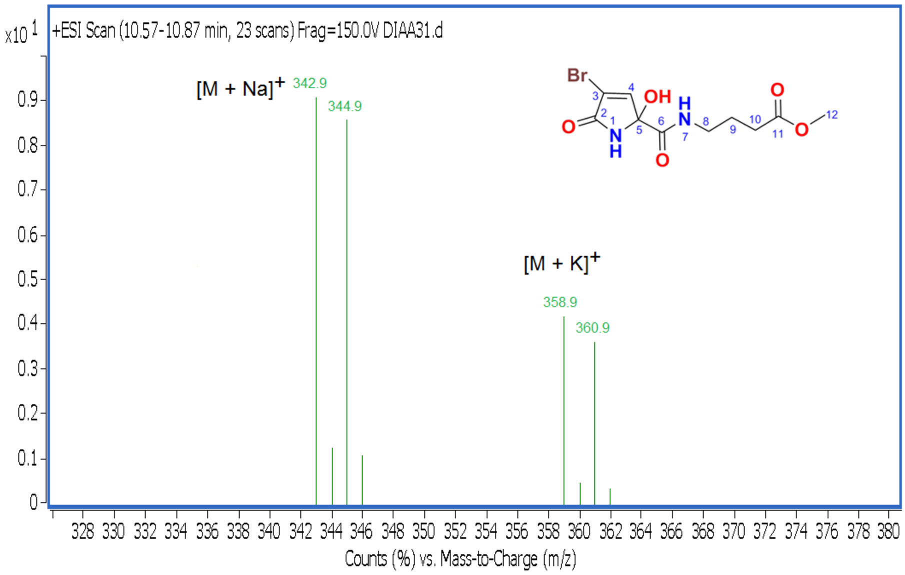

(+)-LRESIMS Spectrum of Compound **2**.

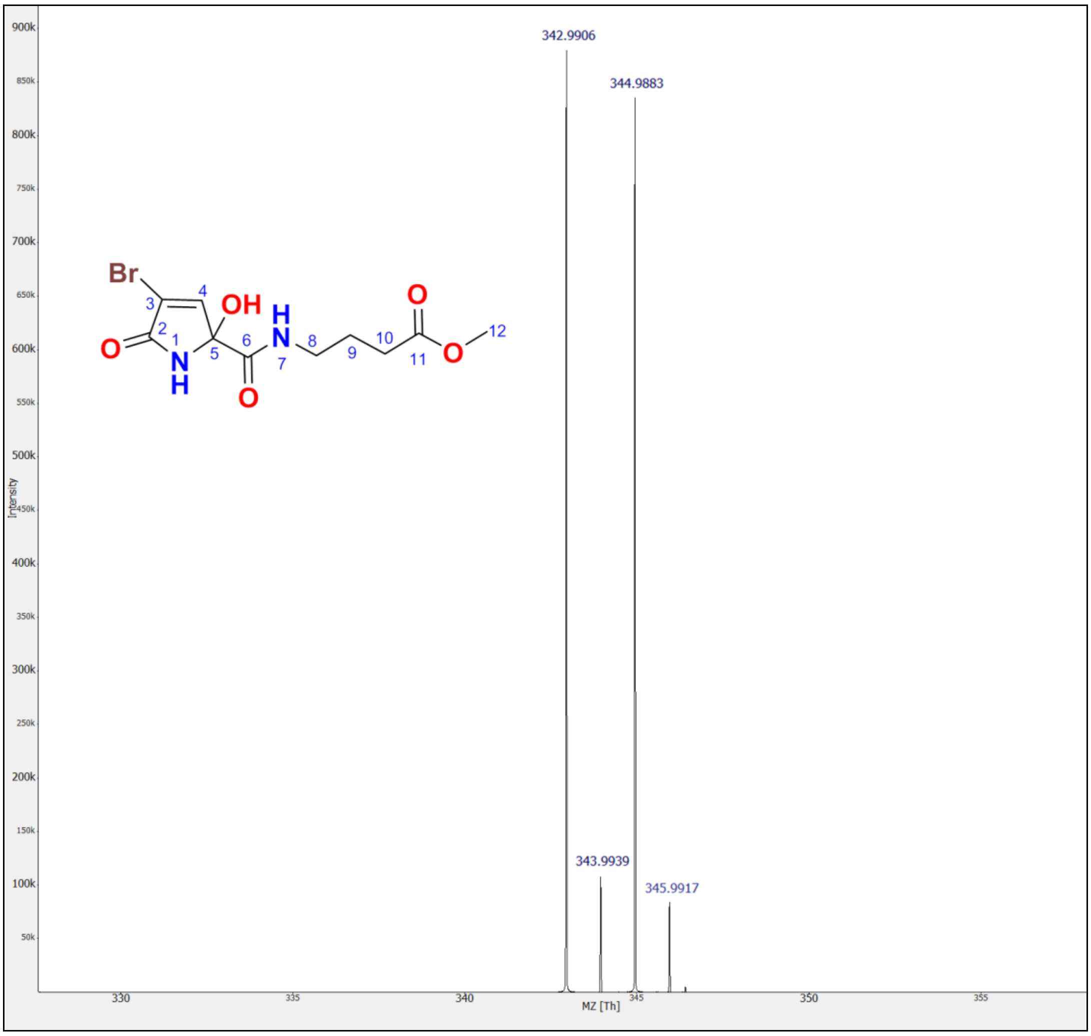

(+)-HRESIMS Spectrum of Compound **2**.

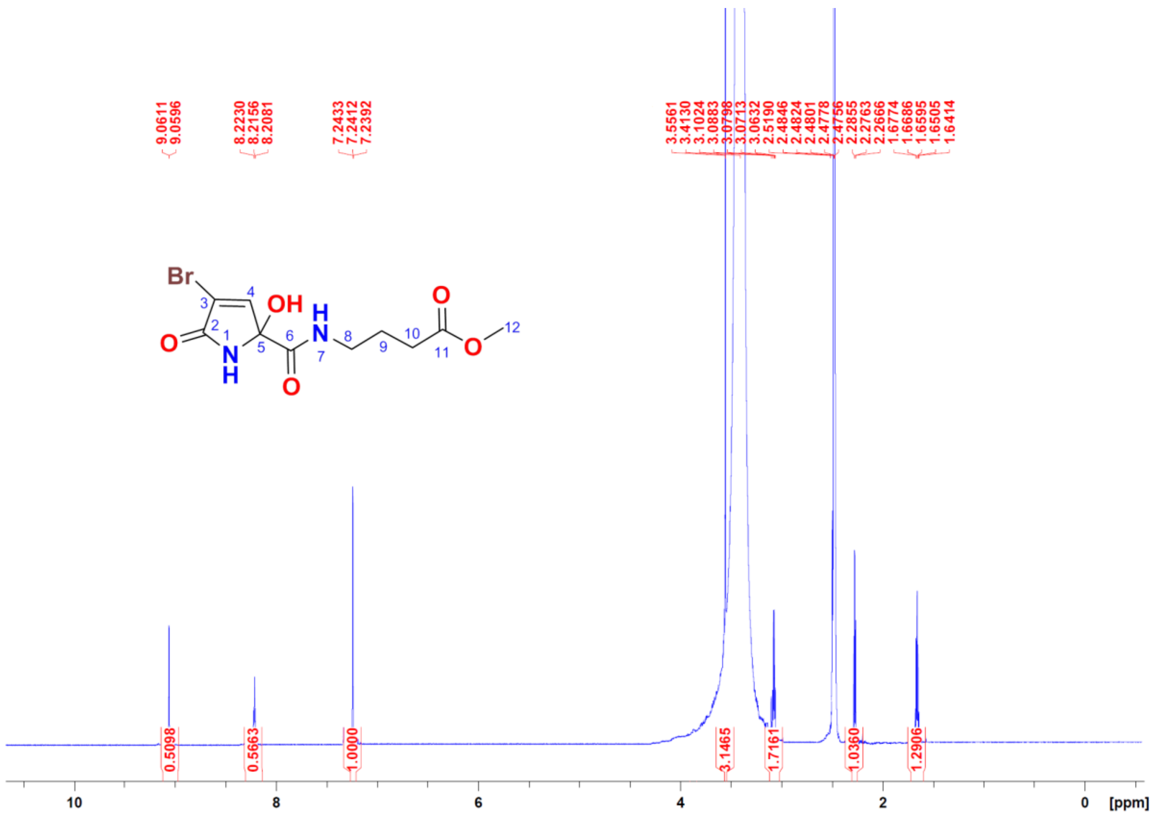

^{1}H NMR Spectrum of Compound **2** (DMSO-d_6).

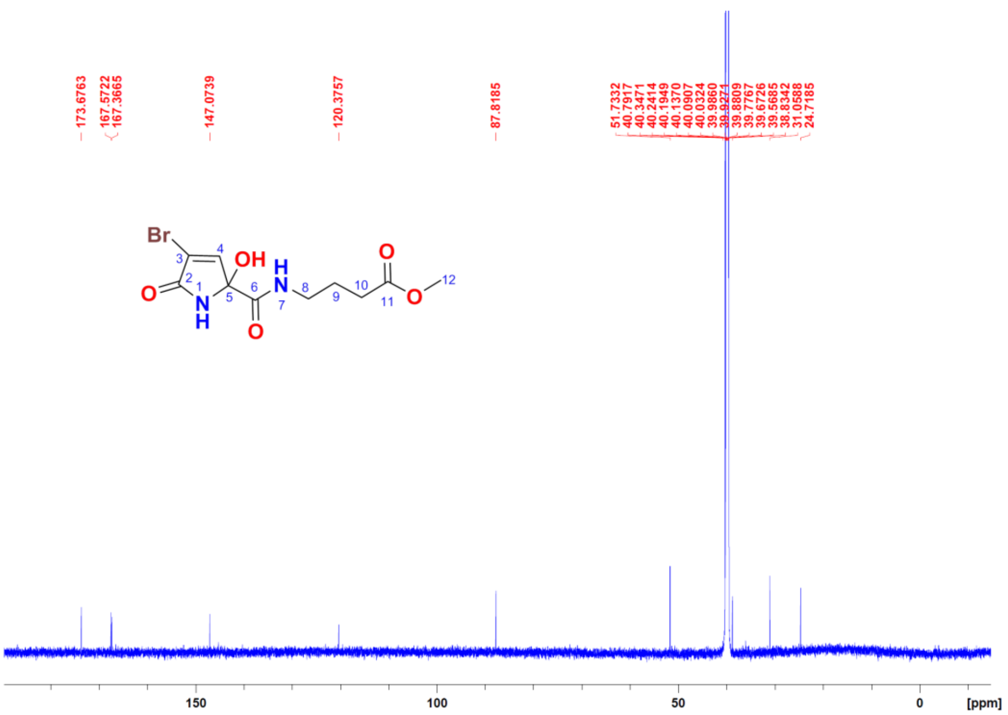

^{13}C NMR Spectrum of Compound **2** (DMSO-d_6).

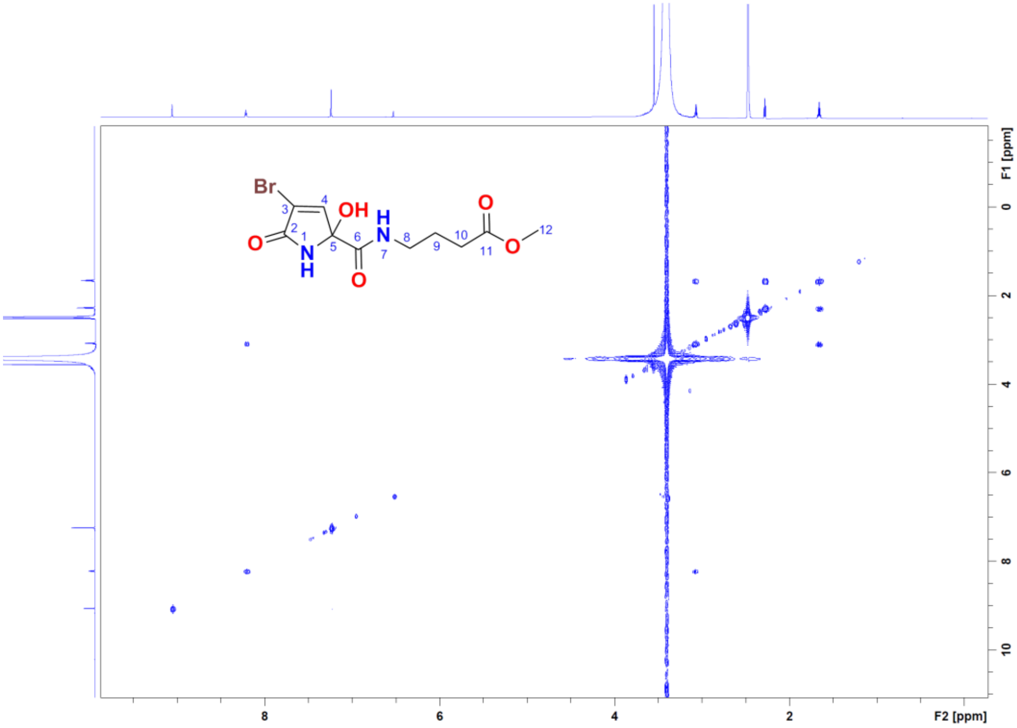

^{1}H-^{1}H COSY NMR Spectrum of Compound **2** (DMSO-d_6).

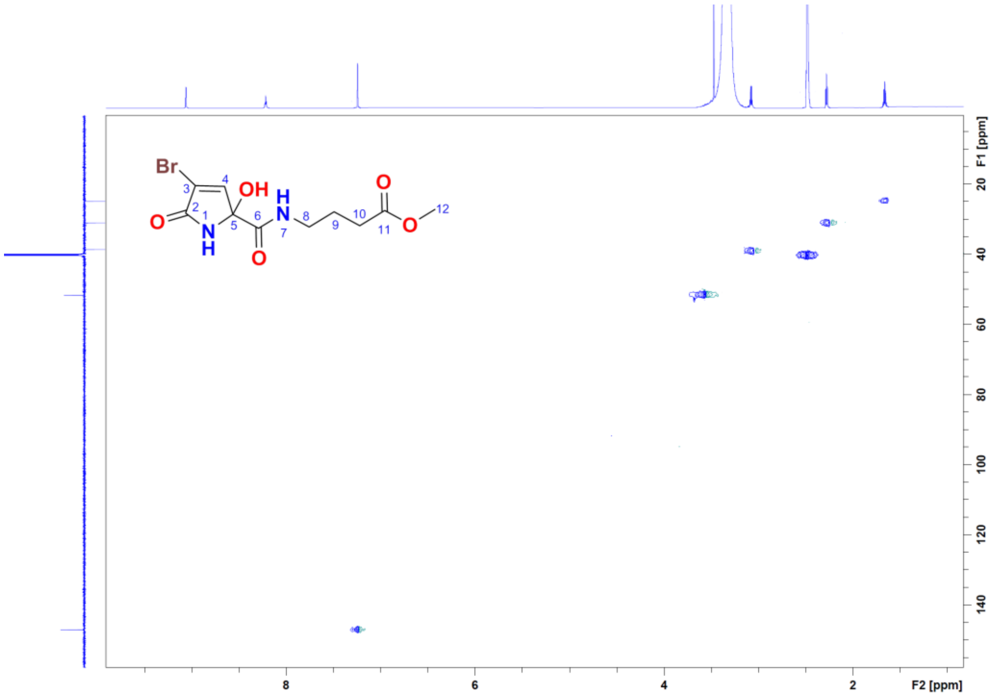

HSQC Spectrum of Compound **2** (DMSO-d_6).

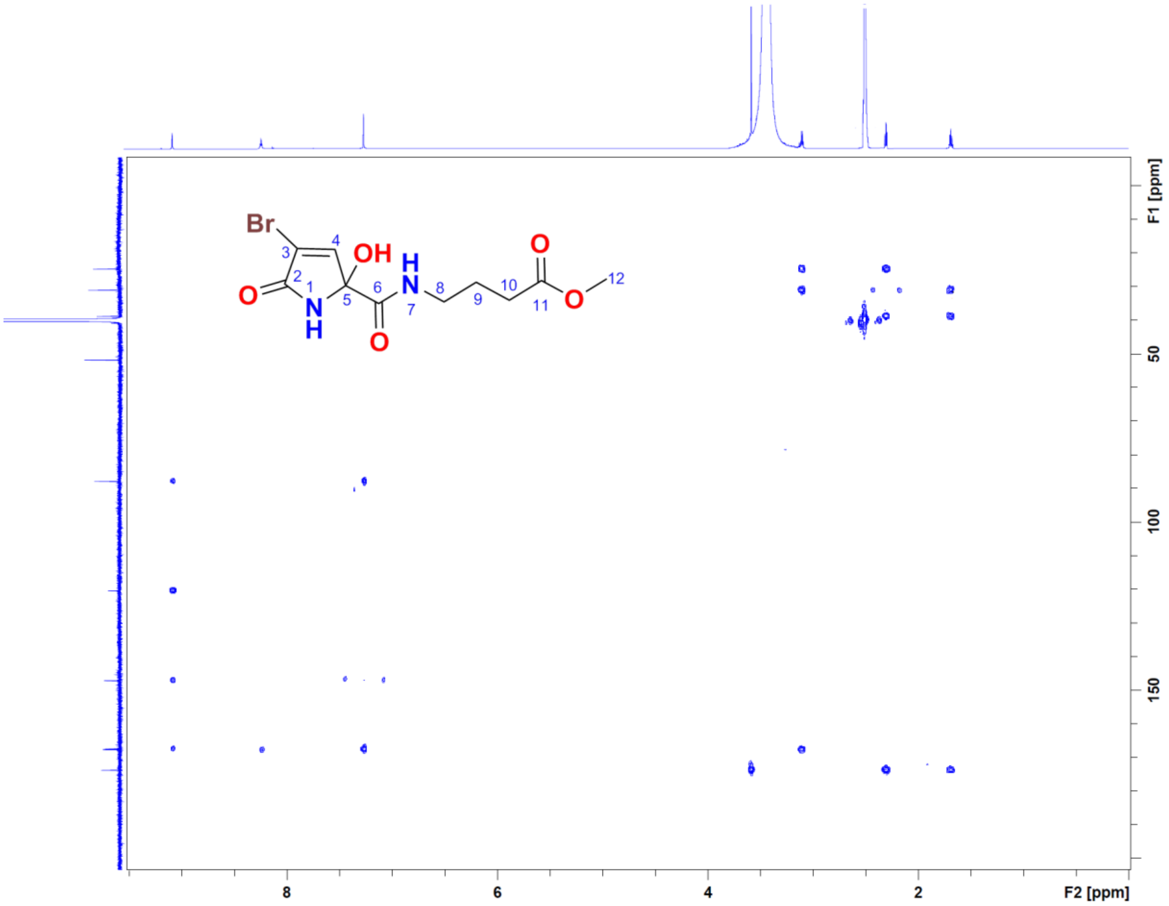

HMBC Spectrum of Compound **2** (DMSO-d_6).

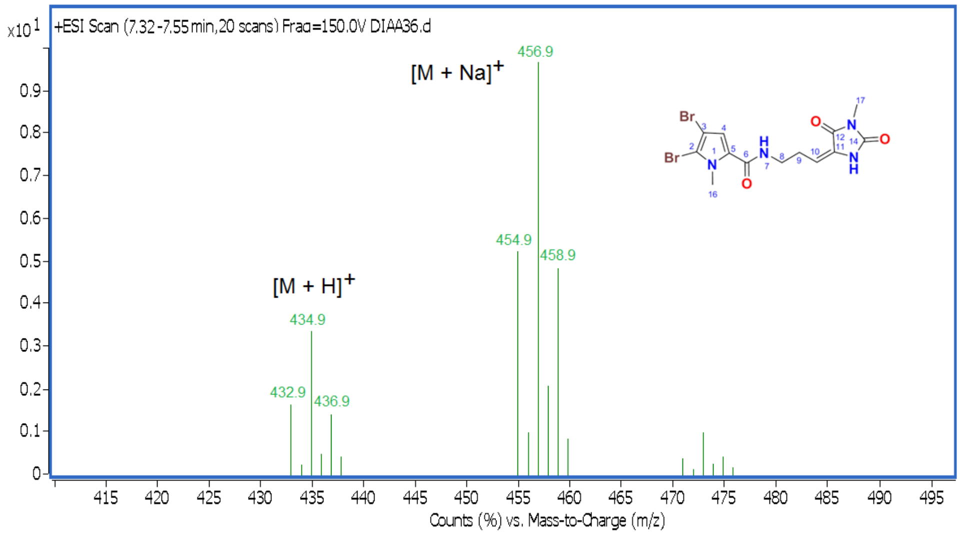

(+)-LRESIMS Spectrum of Compound **3**.

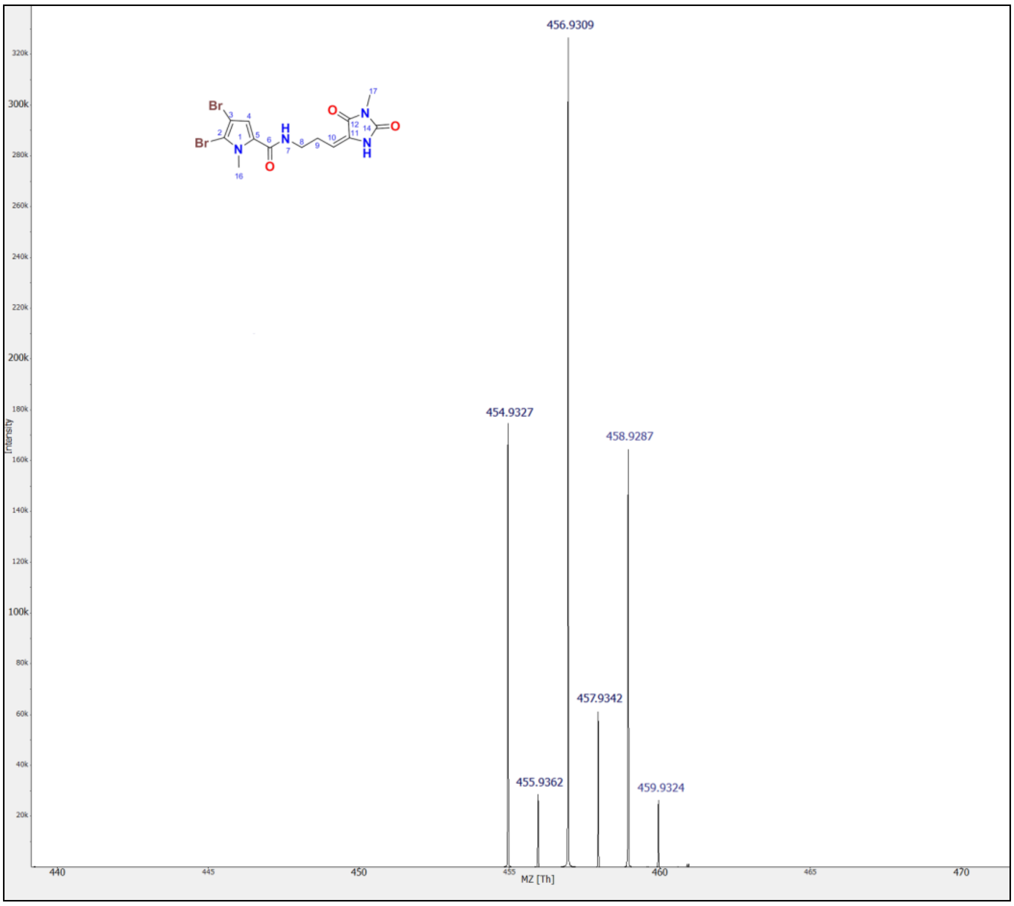

(+)-HRESIMS Spectrum of Compound **3**.

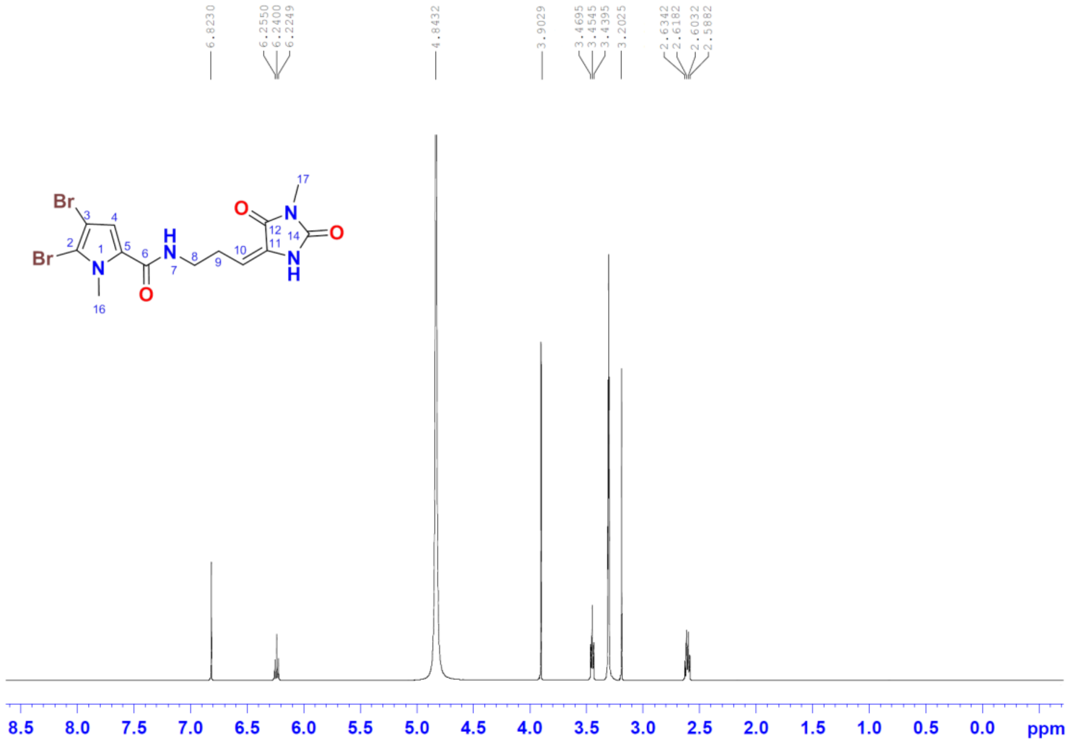

^{1}H NMR Spectrum of Compound **3** (CD_3OD).

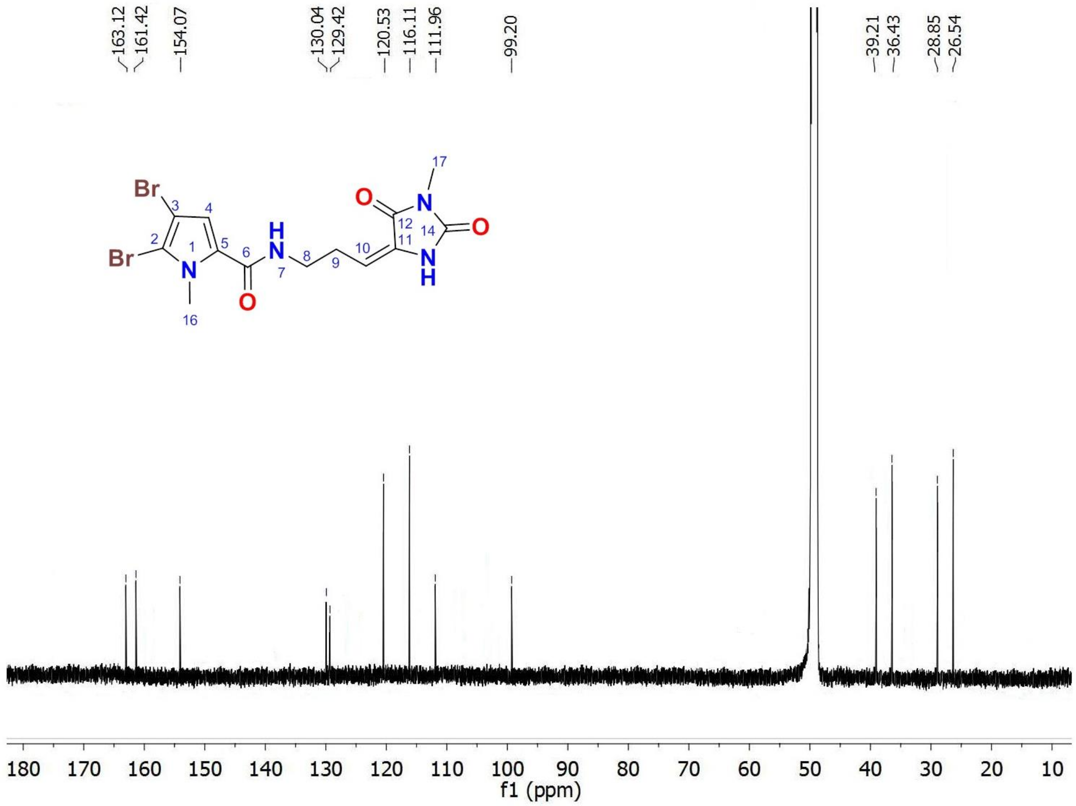

^{13}C NMR Spectrum of Compound **3** (CD_3OD).

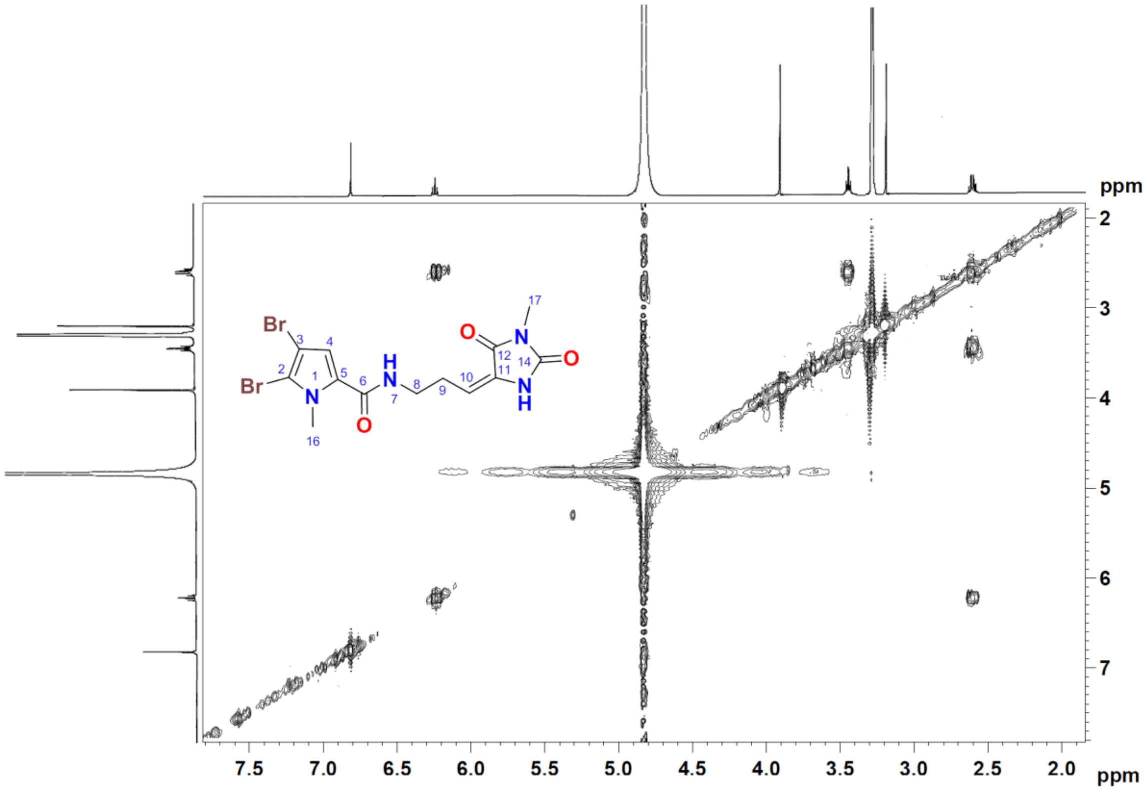

^{1}H-^{1}H COSY Spectrum of Compound **3** (CD_3OD).

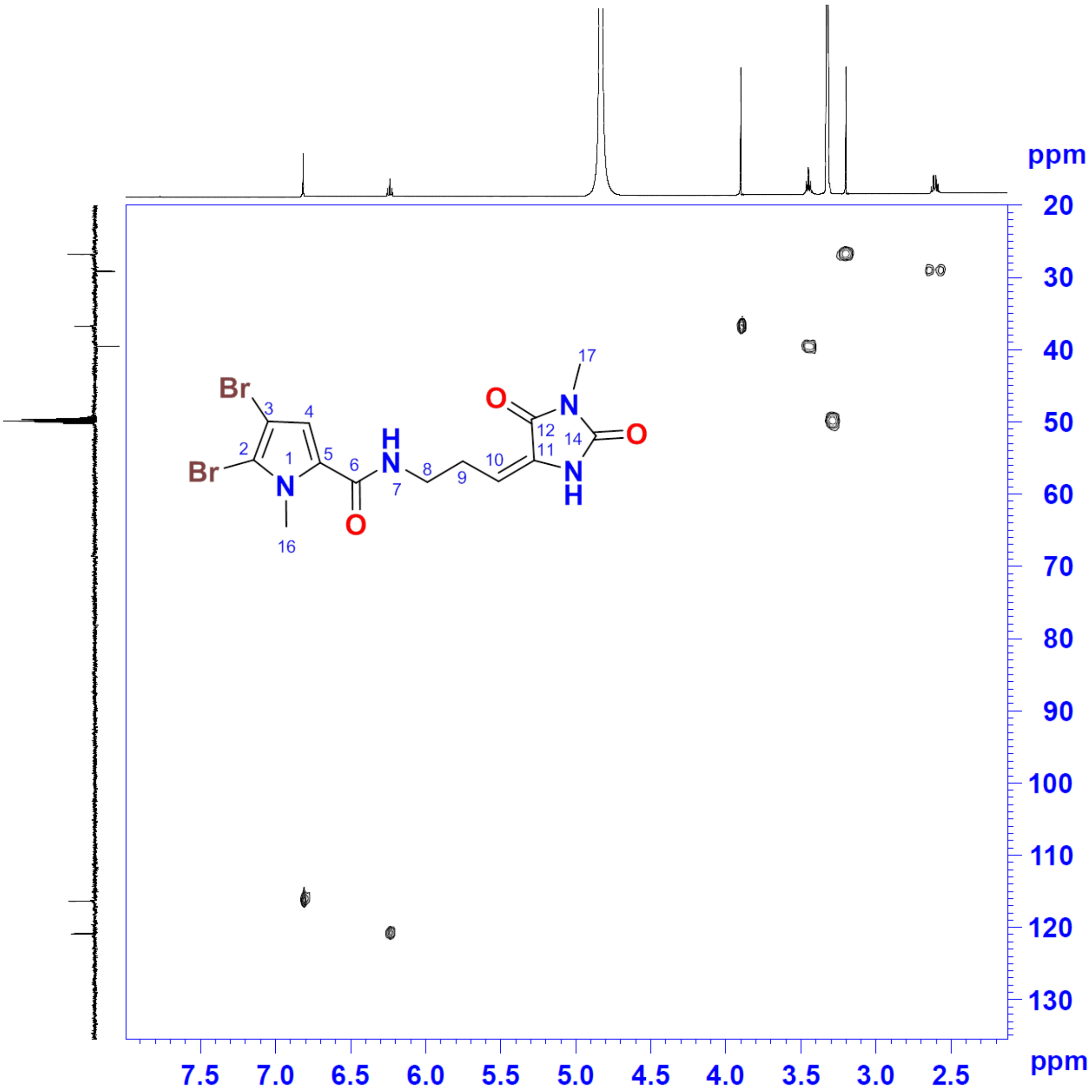

HSQC Spectrum of Compound **3** (CD_3OD).

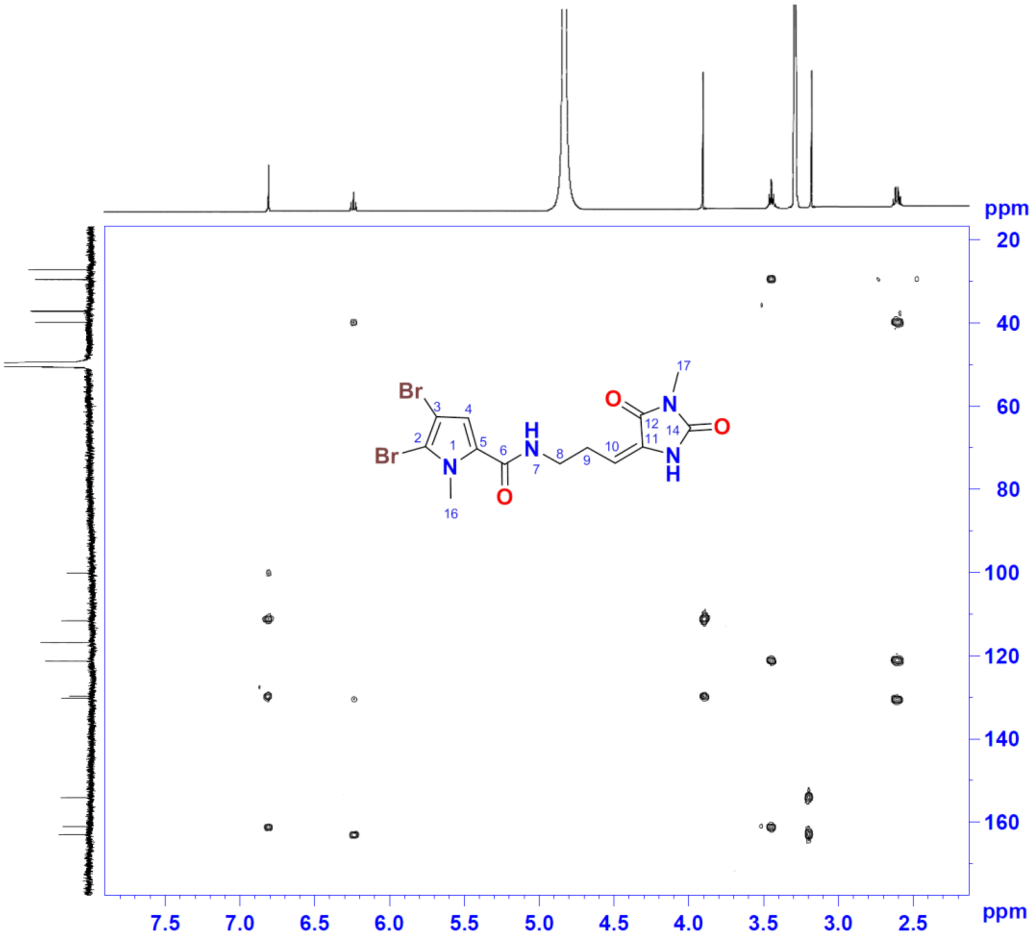

HMBC Spectrum of Compound **3** (CD_3OD).

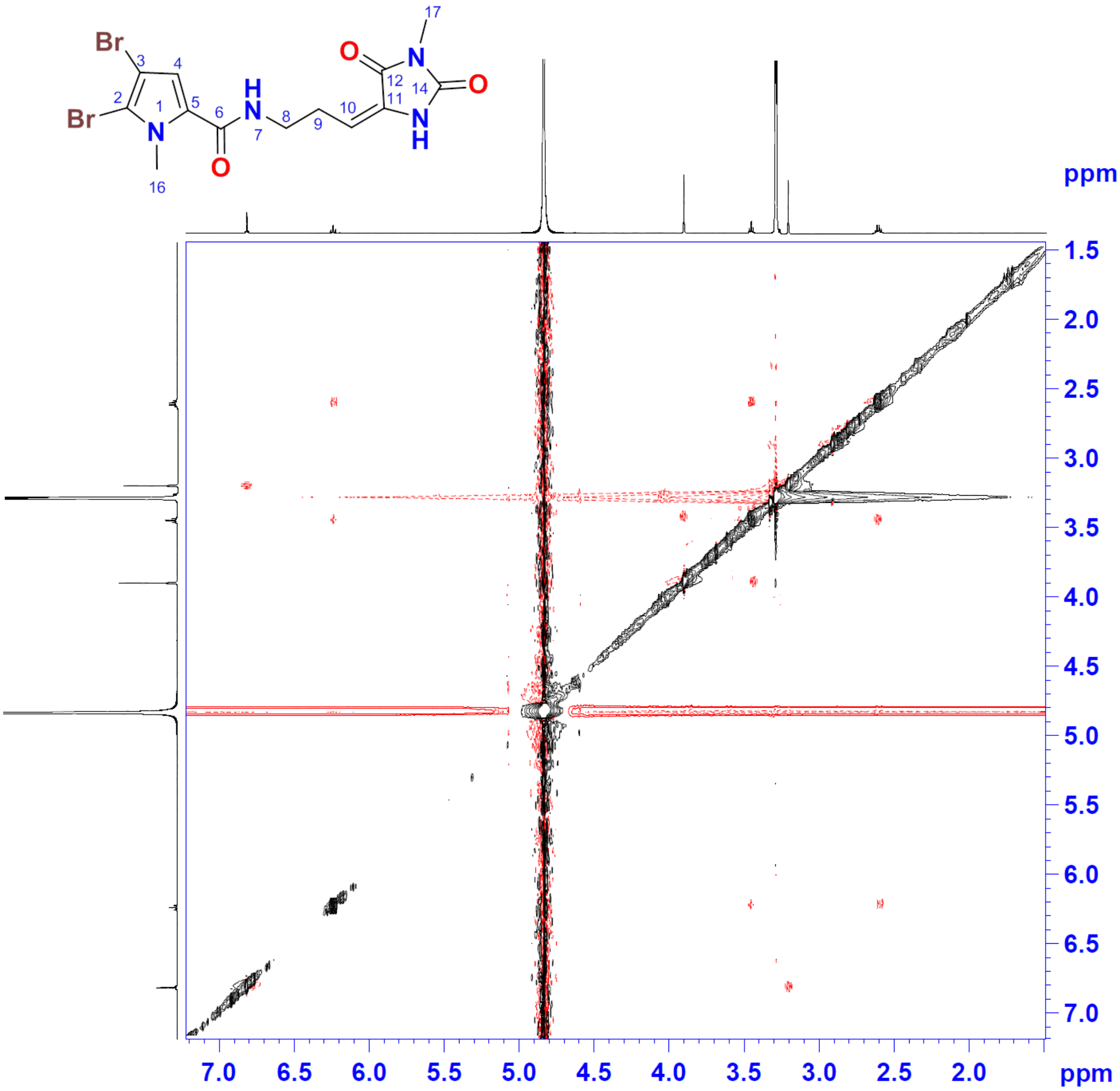

^{1}H-^{1}H NOESY Spectrum of Compound **3** (CD_3OD).

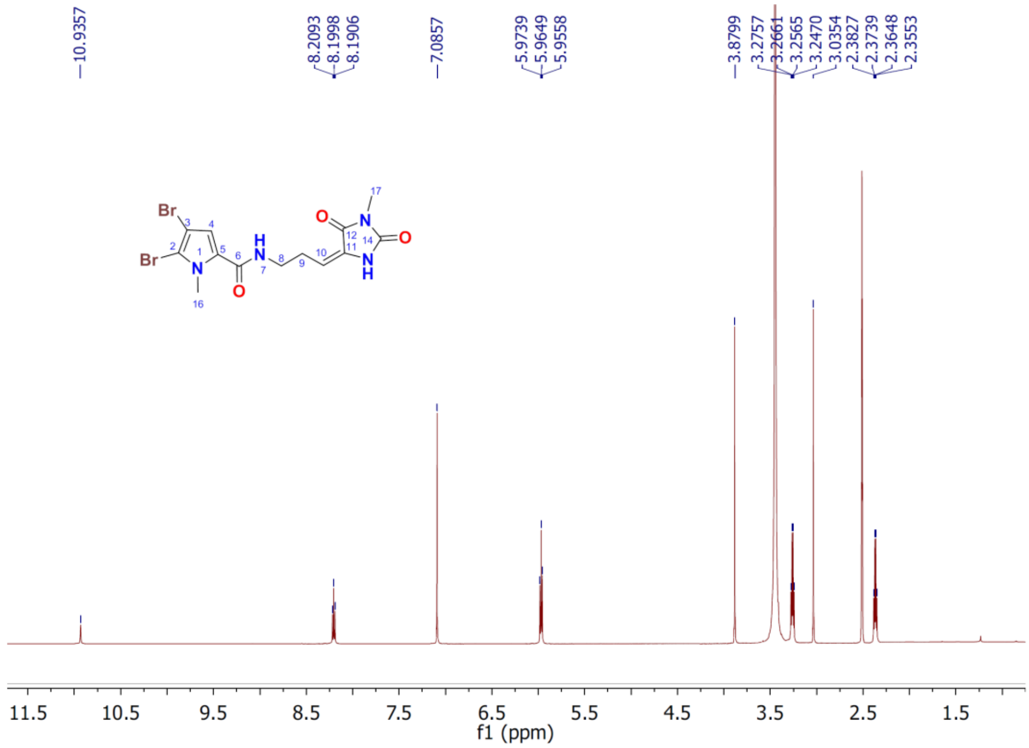

^{1}H NMR Spectrum of Compound **3** (DMSO-d_6).

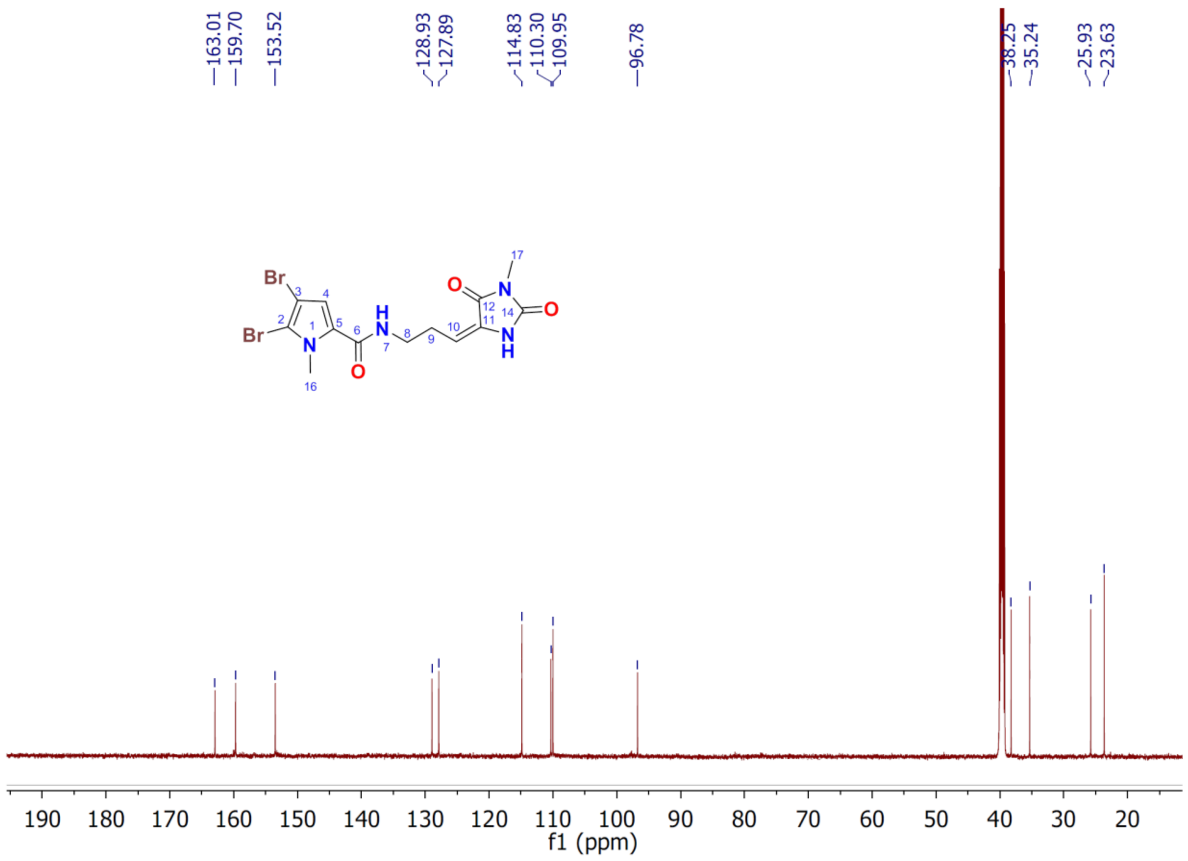

^{13}C NMR Spectrum of Compound **3** (DMSO-d_6).
